# Effect of Carbonaceous Contaminants with Different Degrees of Carbonization on the Wear of 42CrMo4 Steel Under Boundary Lubrication

**DOI:** 10.3390/ma19204416

**Published:** 2026-10-20

**Authors:** Andrzej N. Wieczorek, Iwona Jonczy, Krzysztof Filipowicz, Mariusz Kuczaj, Arkadiusz Pawlikowski, Marcin Staszuk, Dariusz Łukowiec, Anna Kasprzyk

**Affiliations:** 1 Faculty of Mining, Safety Engineering and Industrial Automation, Silesian University of Technology, Akademicka 2 Street, 44-100 Gliwice, Poland; iwona.jonczy@polsl.pl (I.J.); krzysztof.filipowicz@polsl.pl (K.F.); mariusz.kuczaj@polsl.pl (M.K.); arkadiusz.pawlikowski@polsl.pl (A.P.); 2 Faculty of Mechanical Engineering, Silesian University of Technology, Konarskiego 18A, 44-100 Gliwice, Poland; marcin.staszuk@polsl.pl; 3 Materials Research Laboratory, Faculty of Mechanical Engineering, Silesian University of Technology, Konarskiego 18A, 44-100 Gliwice, Poland; dariusz.lukowiec@polsl.pl; 4 Refractory Materials Division in Gliwice, Institute of Ceramics and Building Materials, Łukasiewicz Research Network, Toszecka 99, 44-100 Gliwice, Poland; anna.gerle@icimb.lukasiewicz.gov.pl

**Keywords:** wear, coal, claystone, tribology, wear-resistant steels

## Abstract

The aim of this study was to analyse the quantitative and qualitative effects of mineral oil contamination in the form of crushed hard coal and coal shale particles on the tribological wear of 42CrMo-4 steel under boundary lubrication conditions. The tests were conducted using a roller-on-roller tribological test rig, and the surfaces were analysed by SEM, XRF, EDS, XRD, Raman spectroscopy, and profilometry. An analysis of the impact of carbon and clay contaminants in mineral oil on the wear of 42CrMo-4 steel showed that the degree of carbonization of the added carbon and the mineral fraction content jointly influenced the thermochemical behaviour of the contaminants and the intensity of surface wear. Variant 1, containing carbon with a medium degree of carbonization and low mineral content, exhibited the lowest wear, with a spalling depth of approximately 10 µm—about 25% of the values observed for the other variants—and a wear rate approximately 12.5 times lower than that of the other variants. The favourable may be associated with carbon plasticization and the formation of a protective carbon-rich layer that limited direct interaction between surface asperities. In Variants 2–5, most likely formed more brittle transformation products that did not provide comparable surface protection, resulting in deeper spalling. EDS analysis revealed carbon-rich particles on the surface tested under Variant 0 conditions, indirectly supporting the possibility of thermal oil degradation and high local contact temperatures.

## 1. Introduction

42CrMo-4 steel is one of the most commonly used structural steels for heat treatment in heavy industry, particularly in mining. Due to its favourable combination of high strength, fatigue resistance, and good hardenability, it is widely used in machine components exposed to significant mechanical loads and intense tribological stresses. In mining, 42CrMo-4 steel is commonly employed for shafts, chain drums, gears, and other components of power transmission systems. In particular, in gearboxes with a power rating of up to approximately 100 kW, this steel is frequently used for gears after induction hardening. This treatment increases contact load capacity and wear resistance. However, under the harsh operating conditions of mining machinery—such as variable loads, high slip, or lubrication with oil contaminated with solid particles—this solution is often insufficient, and components made of this steel are prone to intense tribological wear. Understanding the mechanisms of these processes is essential for reducing component wear, extending service life, and decreasing the demand for materials and energy in mining operations, thereby limiting their environmental impact.

**Figure 1:**
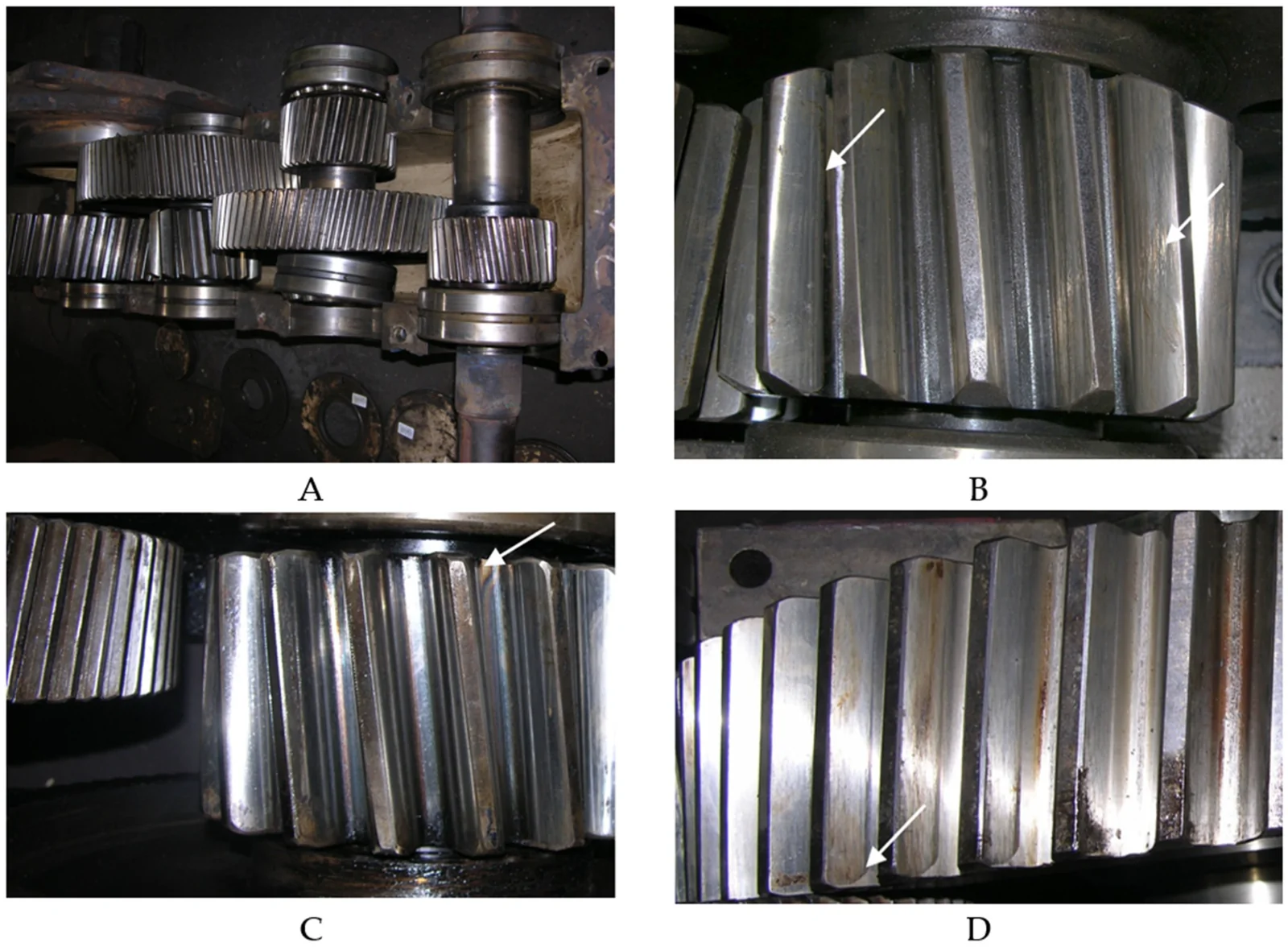
Belt conveyor gearbox with gears made of 42CrMo4 steel after operation under mining conditions: (**A**) general view, (**B**) surface scratches on the gear teeth, (**C**) local tooth surface overheating and spalling, and (**D**) abrasive wear of the tooth flank. The arrows indicate the locations of gear damage. Photographs from the authors’ collection.

Wieczorek and Pawlikowski [1] demonstrated that the commonly used gearbox in belt conveyors with a power transmission of 100 kW, equipped with 42CrMo-4 steel gears that have undergone induction hardening, operates in a boundary and mixed friction regime, which significantly contributes to the development of damage caused by improper lubrication. Figure 1 shows typical surface damage for this gearbox, including scratches, tooth overheating, and abrasive wear.

Over the course of operation of machines and equipment, lubrication conditions may change due to the impact of the working environment, e.g., by way of a change in instantaneous load or operating temperature. Changes in operating conditions may also be continuous in nature on account of the time-dependent degradation of oil properties. The effects of oil degradation may include the following: colour change, increased acid number, formation of deposits and lakes, increased content of solid particles (solid contaminants), water ingress into oil, viscosity change, etc. [1,2,3,4,5].

Lubrication of friction nodes in driven machines [6,7,8,9] with contaminated oil typically renders them non-operational prematurely. Impure oil usually contains particles such as mineral grains, metal swarf, or water. The aforementioned contaminants can damage machine components, especially their drive units, leading to progressive wear and even failure. Such components include gear wheels, bearings, and seals, in particular. As for abrasives containing very hard grains, damage occurs as a consequence of micro-scratching or micro-ridging. The effects of such wear processes will depend significantly on the size, type, and hardness of the oil-contaminating particles. The adverse effect of hard mineral abrasives on the wear of machine components is well-known and extensively described in the literature on the subject [10,11,12,13,14]. Contaminated oil can also deteriorate in terms of its capacity to lubricate parts properly, resulting in excessive friction, overheating, and reduced performance. In many industries, degradation processes caused by lubrication with contaminated oil are counteracted by filtering [15,16,17,18,19], and fresh oil is added or replaced, yet in many cases this is still insufficient.

The potential effects of the presence of solid contaminants in lubricating oil depend on momentary lubrication conditions at friction nodes [20,21,22,23,24,25]. The key parameter determining the lubrication conditions is relative oil film thickness λ, defined by the following relationship [26,27,28]:

(1) $$\lambda =\frac{h_{min}}{\sqrt{{Rq}_{1}^{2}+{Rq}_{2}^{2}}}$$

where h_min_—the minimum lubricant film thickness; Rq_1,2_—the root mean square surface finish of contacting bodies, respectively.

**Figure 2:**
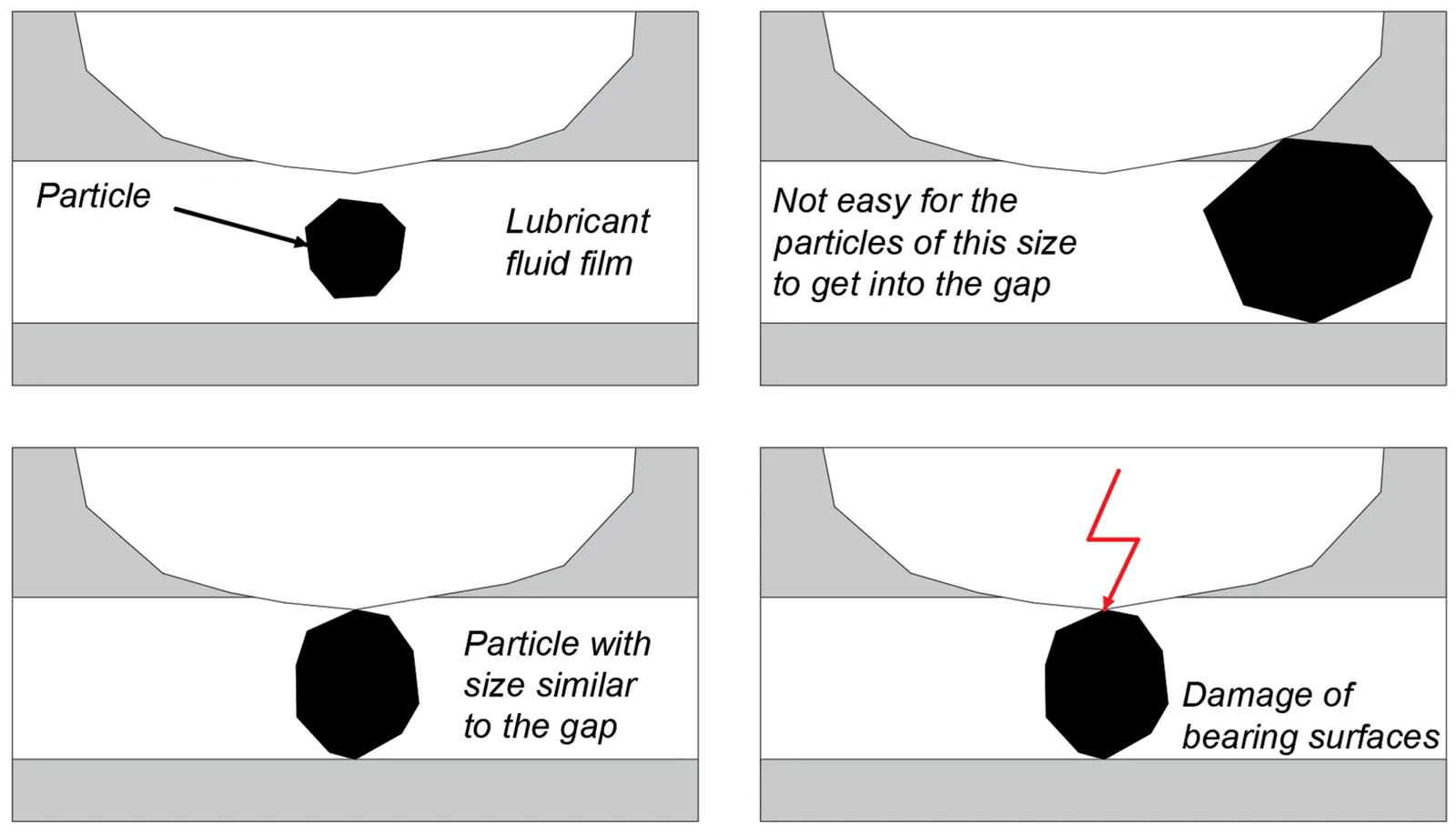
Potential relationships between the minimum oil film thickness and the size of oil contaminating particles; based on [30].

The λ parameter also determines the type of friction occurring between mating surfaces. Lubrication conditions, and so the friction type as well, can be determined based on the λ parameter [21,23,26,27,29] given by formula (1). Within the range of λ = (0.1), boundary friction is to be expected. In the range of relative oil film thickness of λ = (1.3), mixed friction is the dominant type, while the lubrication conditions which develop within the range of λ = <3.10> are characterised by the formation of an oil film with a thickness exceeding that of surface irregularities. Above the relative oil film thickness value of λ > 10, hydrodynamic fluid friction occurs. The fact that damage occurs on the surface depends on the relationship between the minimum oil film thickness and the size of the oil contaminating particles. According to Moon [30], there are three cases (Figure 2) of such relationships:

(a) The abrasive grain is smaller than the oil film thickness and passes through the mating area without contact,
(b) The abrasive grain is larger than the oil film thickness and stops before the mating area,
(c) The abrasive grain is comparable in size to the oil film thickness.

The third case corresponds to a situation where functional surfaces (e.g., bearing races or gear teeth) are subject to abrasive wear. Particles sized similarly to the oil film thickness can penetrate into the contact area where the film thickness is minimal. The load affecting a given surface is transferred to these particles, which become pressed against the mating surface and exert a cutting action. This causes surface degradation, dimensional changes, and the formation of wear products.

The research conducted by Watanabe et al. [31] has confirmed the relationship between oil film thickness, particle size, and the rate of wear of mating surfaces. What the authors found was that the rate of wear depended largely on the film thickness to particle size ratio. The maximum wear rate was observed when this ratio equalled one, meaning that lesser wear occurred when particles were both larger and smaller than the oil film thickness.

**Figure 3:**
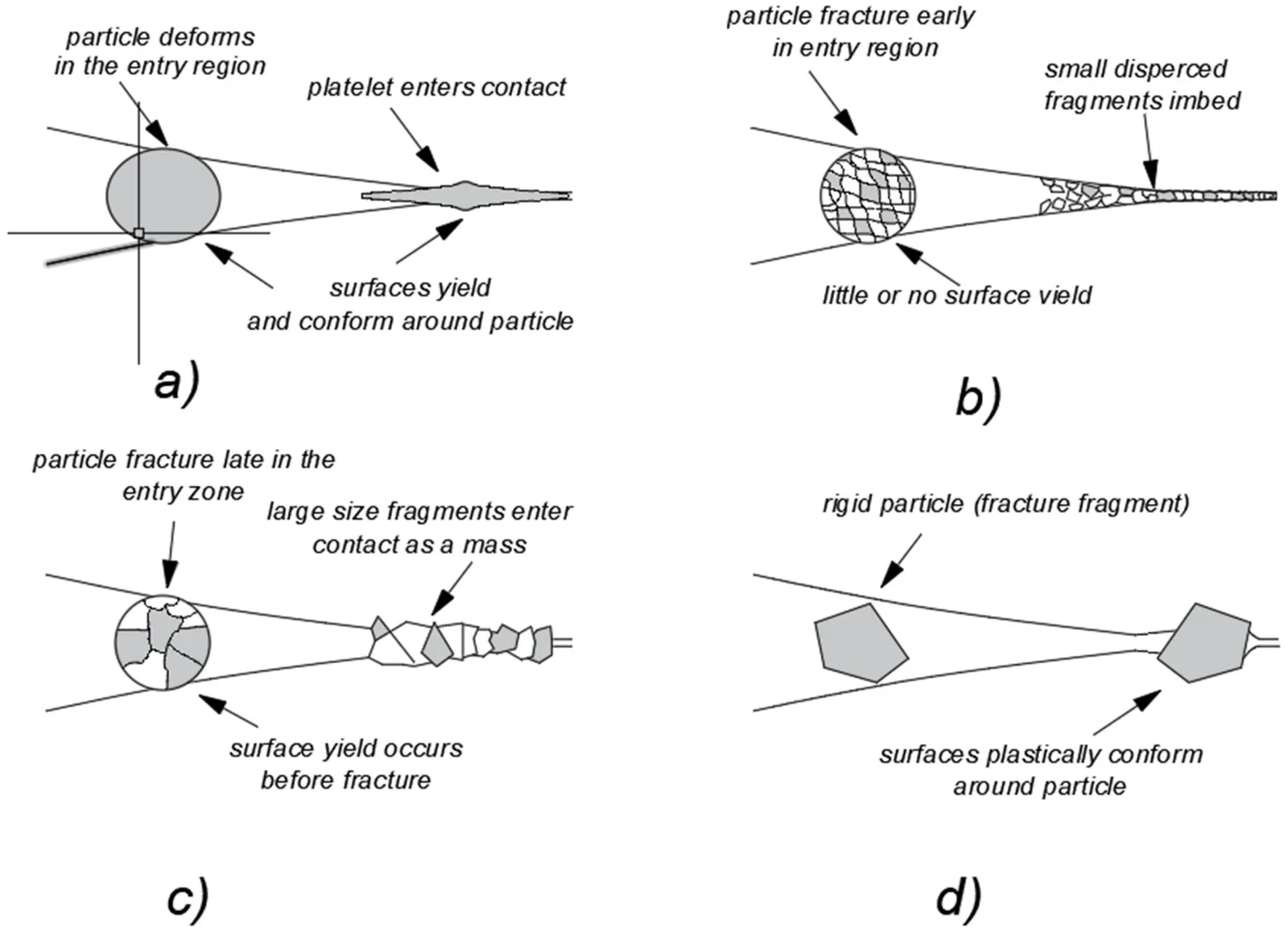
Models of impact of mineral particles in the mating zone of functional surfaces; based on [36].

Dwyer–Joyce et al. [32,33,34] distinguish between four possible behaviour patterns displayed by the mineral particles (Figure 3) which have already entered the mating zone between two functional surfaces:

(a) Plastic abrasive grains deform flat and flatten on mating surfaces,
(b) Abrasive grains consisting of fine mineral fractions become defragmented easily in the mating area (under conditions of lubrication, this may correspond to case a in Figure 2),
(c) Abrasive grains consisting of large mineral fractions become slightly defragmented in the mating area (under conditions of lubrication, this may correspond to cases c and d in Figure 2),
(d) Hard and homogeneous abrasive grains do not become defragmented in the mating area (under conditions of lubrication, this may partly correspond to case b in Figure 2).

The characteristics of oil-contaminating particles depend on the operating environment. Table 1 contains information about the concentration, type, and size of the particles found in lubricating oil, determined by various researchers for different industries.

In the case of abrasives composed of soft rock grains, including coal, some other wear mechanisms may occur, including surface fatigue or delamination processes taking the form of shallow spalling [40,41,42]. However, abrasive mixtures are often inhomogeneous and may contain materials of wear properties that differ significantly. One such material is hard coal, which is very often contaminated with mineral substances and may also contain interlayers of clastic rocks: sandstones, mudstones and argillaceous rocks. Immediately after extraction, hard coal should not be treated as a homogeneous component, but only as a mixture of various minerals and coal.

As mentioned in papers [43,44], the following was determined for non-lubricated friction nodes subject to wear in the presence of hard coal and carbonaceous claystone: the typical forms of damage due to wear mechanisms were micro-scratching and micro-fatigue; there was kaolinite and carbonaceous substance pressed into the scratches caused by surface spalling; metal particles and mineral grains could be partially neutralised by a layer of pressed-in claystone. Wieczorek et al. [45] demonstrated surface damage caused by cutting with mineral fraction grains and flat surface spalling products for coal-mineral and mineral abrasives. Additionally, hard grains were observed to deposit in damaged surface areas, which was facilitated by the presence of carbonaceous fractions. On the other hand, for surfaces worn in the presence of carbonaceous grains alone, wear tests revealed undisturbed traces of the grinding process, suggesting the formation of unstable layers of pressed-in coal. Furthermore, the aforementioned paper introduces two models of wear in the presence of coal and coal-mineral abrasives occurring in a real-life tribological system, namely a scraper moving on the surface of a sliding plate. What has also been determined in that study is the potential impact of the presence of coal-based abrasives on the intensity of the wear process in the components of machinery transporting energy raw materials.

The impact of coal type (lithotype) on abrasive wear was analysed in study [46]. In this respect, slight differences in wear values were found in the coal types subject to tests, and no impact of load on abrasive wear was observed. Following the tests, pressed-in coal grains were observed in the scratches resulting from grinding, and the pressed-in coal also formed layers on the surface of the samples. The resulting coal films exhibited protective and neutralising properties against the destructive action of steel particles.

Report [47] concerning tests of oil–coal mixtures of variable non-carbon fraction content, grain size, and solid contaminant concentration reads that the most favourable wear, thermal, and friction properties were established for the pure oil lubrication variant (roller-block tester) and the lowest friction coefficient values (four-ball tester). The following has also been noted: an increase in the proportion of non-carbon fractions in solid lubricant contaminants, regardless of their particle size, significantly increased the wear of the test samples; as the size of the coal particles contained in the lubricating mixtures increased, the values of the parameters characterising friction and wear decreased; the presence of additional solid particles in the lubricating oil may, to a certain limited extent, improve resistance to scuffing.

In paper [48], on the other hand, it has been demonstrated that gear transmissions for mining applications are operated under unfavourable boundary and mixed lubrication conditions, and the fraction of solid contaminants in their lubricating oil after 1 month comes to 0.4% at the least. Studies of the oil contaminants found in transmissions used in hard coal mines [49] showed that the diameter of the contaminating particles ranged from 0 to 10 μm (an average particle size being approx. 2.5 μm) and that these particles were composed mainly of coal, SiO_2_ and iron particles.

Ostrikov et al. [50] observed in their study that the addition of carbon black significantly improved the lubricity of mineral oil, enhancing its properties, while it activated wear processes in oil without additives. The lubricity of pure oil with additives increased by more than 30% once it had been exposed to ultrasounds, which resulted from the activation of its dispersion medium. It was further noted that small amounts of engine fuel combustion products which penetrated oil increased the lubricity of oil enriched with additives, similarly to other carbon-based additives.

In paper [51], on the other hand, Bölter argues that, as the content of carbon black increases in oil, the coefficient of friction decreases, while surface properties after the wearing-in process deteriorate as the content of carbon black grows. The surfaces of components operated in oils containing carbon black were found to show less damage, and their superficial layers displayed a lower content of anti-wear additives, which may suggest them being bonded by the particles of carbon black.

Pang et al. [52] conducted research on the effects of contamination of solid mining transmission lubricants with coal and rock dust on the wear of steel. Pulverised coal and silica dust with a grain size of approx. 180 µm and 75 µm were used in the experiments. The finding of that study is that wear is considerably dependent on the type of contaminants and their combination, and a small amount of pulverised coal exerts a beneficial effect on account of its lubricating properties, while higher concentrations significantly increase wear. What this study has also demonstrated is the lower coefficients of friction established for oil contaminated with anthracite coal dust (COF = 0.065) compared to oil contaminated with a mixture of coal and silica dust (COF = 0.105). Another conclusion derived from that study is that the cyclic loads attributable to the presence of coal particles lead to dynamic changes in surface stresses in alloy steel, resulting in local stress concentrations which promote initiation of microcracks, and as the friction process progresses, these microcracks propagate, leading to the gradual detachment of material particles and the formation of wear products. The aforementioned study has also revealed that the presence of reactive elements, such as phosphorus (P) and sulphur (S), contained in coal-derived contaminants significantly intensifies surface degradation by accelerating oxidation processes.

In their respective papers, Tlotleng et al. [53], Xia et al. [54], Yarali et al. [55], Shao et al. [56], Shi et al. [57], Terva et al. [58], Wang et al. [59], Labaš et al. [60], Ngoy et al. [61], Wells et al. [62,63], as well as Petrica et al. [64] have confirmed the major abrasive effect of hard coal, leading to considerable wear of mining tools and equipment.

On the other hand, based on the results obtained from a numerical model taking into account both the plastic deformation of particles and the forces generated by grease under pressure, as well as the distribution of the heat attributable to friction, including the values of flash temperature, as it is commonly referred to, Niklas [65] has detected a significant effect of thermal load on the cracking and spalling in the superficial layer of surfaces lubricated with oil contaminated with soft particles. Thermal load is generated by way of the processes of forcing through and compression of mineral material grains over the course of elastohydrodynamic contact, leading to a considerable short-term local temperature increase due to friction (a phenomenon known as flash temperature).

According to the same author [66], the peak flash temperature occurs in less than one millisecond, depending on the rolling and sliding velocity. This phenomenon is most powerful just before the particle enters the central contact zone, which determines the location where the maximum temperature is observed. The distribution of heat between the particle and counterfaces is directly dependent on their thermal properties. Typically, most of the heat is transferred to the surface to which the particle adheres. It has also been noticed that the larger and/or harder the particle, the smaller the temperature difference between the counterfaces. The main factor affecting flash temperature is the particle size, not its hardness. The difference in flash temperature between particles of 5 μm and 20 μm in size, made of the same material, can exceed 1000 °C (Table 2).

Frictional heating generates thermal stresses which, in numerous cases, significantly exceed mechanical stresses [67,68]. This phenomenon raises the risk of surface damage, shifting the high-risk zone of plastic deformation closer to counterfaces. Unlike hard particles, which retain their shape, soft particles flatten, thus increasing the friction surface considerably. For this reason, and on account of the predominance of thermal stresses, soft particles can cause serious damage.

Unfortunately, in many cases, oil contamination cannot be avoided in lubricated friction nodes due to sealing system leaks, the use of vents, and the cyclical processes of heating and shrinking of machine bodies [69]. This is a typical mining situation, encountered particularly in hard coal mines, which, despite the use of advanced materials and heat treatment technologies dedicated to this industry [70,71], leads to critical damage, which manifests itself as pitting or abrasive wear, and consequently to the decommissioning of the affected equipment.

The conducted literature review demonstrated that previous studies have focused mainly on assessing the influence of mineral contaminants and coal dust on the wear processes of machine components operating under lubricated conditions. Both the detrimental effect of hard mineral fractions responsible for abrasive wear and the potential lubricating properties of selected carbonaceous materials have been reported. However, the influence of the carbonization degree of carbonaceous contaminants on the wear mechanisms of steel surfaces operating under boundary and mixed lubrication conditions has not yet been clearly explained. In particular, there is a lack of information regarding the relationship between the carbonization degree of the carbonaceous phase, its thermal transformations induced by flash temperatures occurring within the contact zone, and the formation of tribological layers modifying the wear process. This knowledge gap also concerns the influence of carbonaceous claystones, which constitute natural contaminants associated with energy mineral resources. The significance of the thermal transformation products of coal and carbonaceous claystones in the formation of protective layers and in the degradation of the steel surface layer has not yet been determined. Furthermore, the literature lacks models describing the relationship between the carbonization degree of carbonaceous contaminants, their thermal transformations within the friction zone, and the wear intensity of machine components lubricated with mineral oil. Therefore, the novelty of the present study lies in determining the influence of the carbonization degree and mineral matter content of carbonaceous contaminants on the wear mechanisms of 42CrMo4 steel lubricated with mineral oil under boundary lubrication conditions, as well as in explaining the role of the thermally transformed carbonaceous phase in the formation of tribological layers responsible for reducing or intensifying surface degradation.

## 2. Materials and Methods

**Figure 4:**
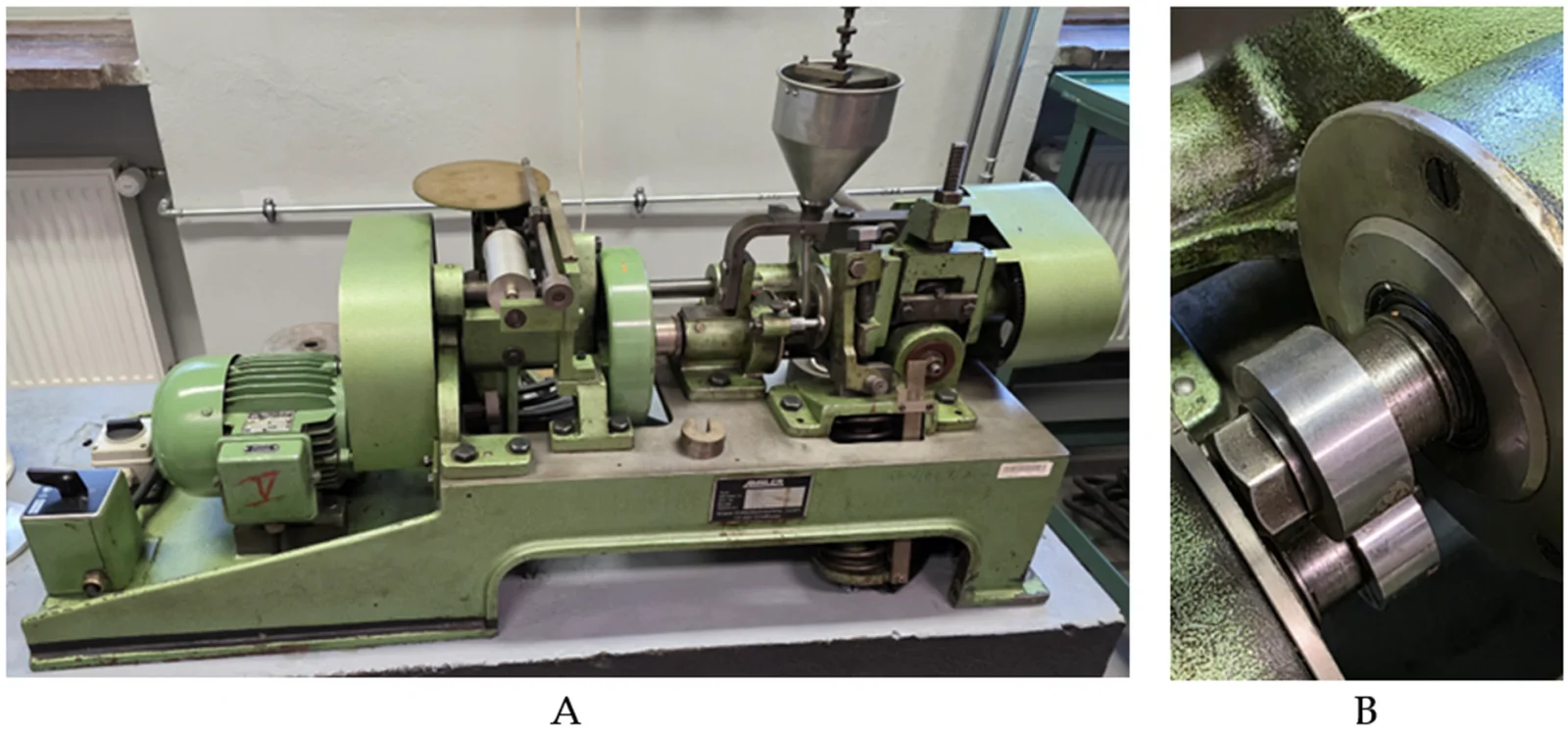
Test rig used in the wear experiments: (**A**) view of the test stand; (**B**) view of the test head with roller specimens.

Tests enabling quantitative and qualitative determination of wear of mating elements were conducted at an Amsler roller-type test rig [72]. The test assembly consisted of two cylindrical rings contacting each other with their cylindrical surfaces, loaded with a constant radial force, and rotating in such a way as to cause counter-motion of the samples. Figure 4 shows the test rig used in the wear tests, including a view of the roller-on-roller test stand and a view of the test head with roller specimens.

The wear tests at the roller test rig proceeded under the following conditions:

– Friction type—rolling with slide,
– Load—constant radial force of F = 490 N,
– Sample contact area width—b = 10 mm,
– Rotational speed—n_1_ = 200 min^−1^,
– Sample circumferential speed—v_p_ = 0.8 m·s^−1^,
– Counter-sample circumferential speed—v_pp_ = 0.92 m·s^−1^,
– Counter-motion of samples.

In this study, mass loss was adopted as the primary parameter characterising the wear of the samples and was determined according to the following equation:

(2) $$∆M=\left(M_{ILS}-M_{ELS}\right)+\left(M_{IUS}-M_{EUS}\right)$$

where ΔM—Mass loss, M_ILS_—initial mass of the lower sample, M_ELS_—mass of the lower sample after the test, M_IUS_—initial mass of the upper sample, and M_EUS_—mass of the upper sample after the test.

Based on the measured mass loss, the volume loss was also calculated (Equation (3)), which subsequently allowed the wear rate to be determined (Equation (4)) [73].

(3) $$∆V=\frac{∆M}{D}$$

where ΔV—volume loss, D—density.

(4) $$WR=\frac{∆V}{S}=\frac{∆V}{\pi \cdot d\cdot n\cdot t}$$

where WR—wear rate, S—sliding distance, d—diameter, n—rotational speed, t—time.

The samples and counter-samples were made of the 42CrMo-4 steel (quenched and tempered at a low temperature of 160 °C) with a surface hardness of 54 ± 2 HRC. The chemical composition of the steel was determined using an SPECTRO spark optical emission spectrometer (SPECTRO Analytical Instruments GmbH, Kleve, Germany). Three measurements were performed, and the measurement uncertainty was calculated using Student’s t-distribution at a 95% confidence level. The chemical composition is provided in Table 3. After the heat treatment, this steel displayed a structure of tempered martensite. Following the heat treatment, the samples were ground to achieve the required dimensional accuracy and a uniform surface roughness.

Both the samples and counter-samples were of identical dimensions: outer diameter of ϕ40, hole diameter of ϕ16, and sample width of 10 mm. Mass loss measurements were performed after 5, 10, 15, 30 and 60 min of co-operation of the rings tested. Before the wear test began and after each friction cycle, the mass of the sample (having been carefully cleaned and dried) was determined five times using an analytical balance characterised by a measurement accuracy of 0.1 mg. Student’s t-test was used to determine the measurement uncertainty of the samples at a confidence level of 0.95. Each measurement was performed in three replicates.

Pure VG 220 mineral oil and 5 oil mixtures with 1% mass fraction of the coal and claystone particles examined (these minerals have been described in detail in Section 3.1 of the paper) were used in the tests. The oil–coal and oil–claystone mixture variants taken into account have been listed in Table 4. Table 5 provides a compilation of the parameters characterising pure oil and, due to the very similar results obtained, the average viscosity values obtained for Variants 1 ÷ 5 of the mixtures was examined. In the study, grain fractions of coal and claystone passing through a 100 × 100 µm sieve were used. The average grain size was determined based on measurements of particles visible in microscopic analysis (highly fragmented fractions with sizes below 1 µm were not considered).

**Figure 5:**
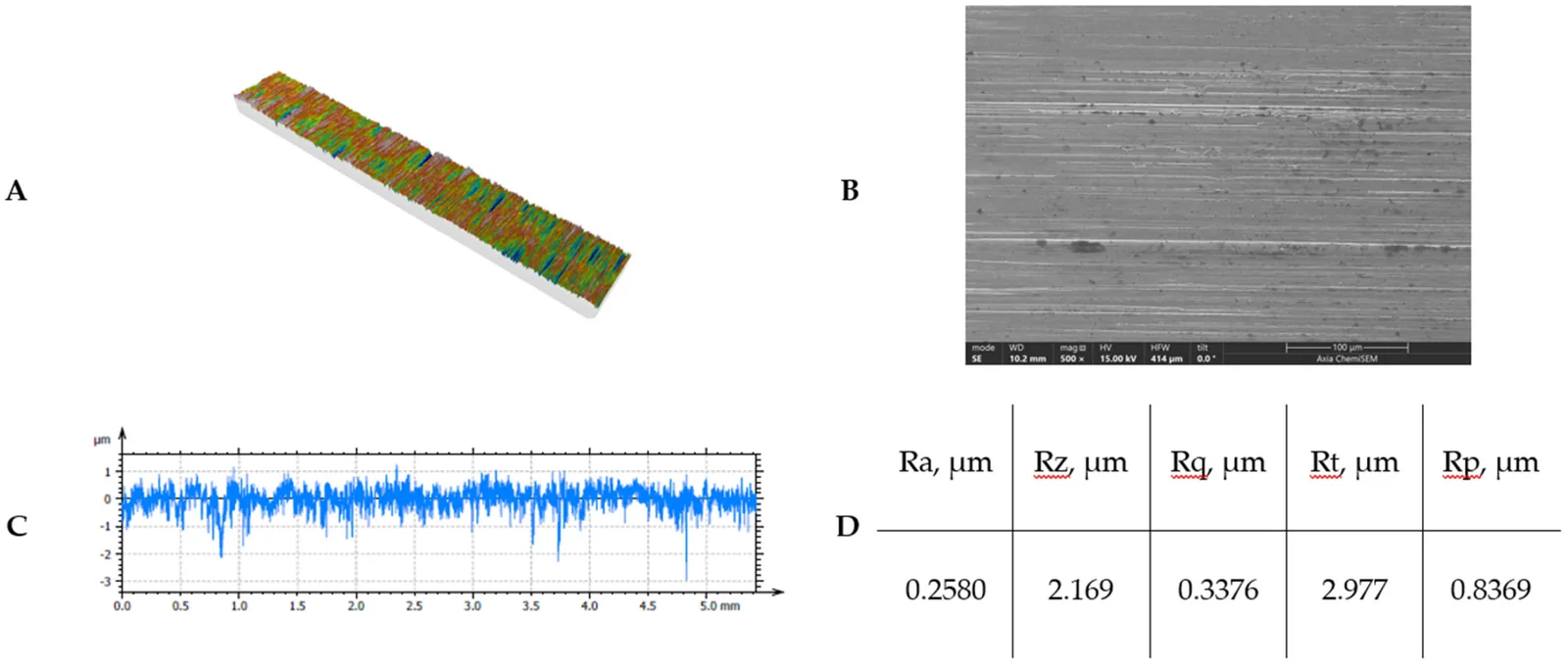
Results of surface roughness measurements: (**A**) 3D view of the surface, (**B**) profilogram, (**C**) SEM view of the surface, (**D**) surface roughness measurement results.

Additionally, in order to characterise the conditions of lubrication of the test samples, the following was determined:

– Surface roughness (measurement results provided in Figure 5);
– Oil film thickness; the calculation results obtained for the roller–roller combination have been provided in Table 6;
– Hertzian surface pressures and the location of the maximum pressure point (calculation results compiled in Table 7).

To determine the minimum lubricant film thickness *h*_min_, the calculation methodology described in ISO/TR 15144-1:2014 (E) [74] was applied. This standard is intended for estimating the oil film thickness in cylindrical gear contacts. The basic relationship used in the calculations was as follows:

(5) $$h_{min}=1600\cdot \rho_{n,Y}\cdot G_{M}^{0.6}\cdot U_{Y}^{0.7}\cdot W_{Y}^{-0.13}\cdot S_{GT,Y}^{0.22}$$

where ρ_n,Y_—the normal radius of relative curvature at local point Y, G_M_—the material parameter, U_Y_—the local velocity parameter, W_Y_—the local load parameter, and S_GF,Y_ is the local sliding parameter.

Based on the lubricating film thickness calculations, one can conclude that the ring–ring friction pair tested was subject to boundary friction conditions.

The chemical composition of the coal abrasives was determined by X-ray fluorescence (WD-XRF) using a Rigaku ZSX Primus II WD-XRF spectrometer (Rh lamp) (Rigaku Corporation, Tokyo, Japan).. The samples were dried, and then the relevant loss on ignition was determined at the temperature of 1025 °C. Roasted to obtain a constant mass, the samples were melted using an off-the-shelf mixture of lithium tetraborate, lithium metaborate, and lithium bromide (66.67%, 32.83%, and 0.5%, respectively) characterised by a flux purity suitable for XRF (from Spex). The spectrum was analysed qualitatively by identifying spectral lines and determining their possible coincidences. On such a basis, analytical lines were selected. Semi-quantitative analysis was performed using the SQX Calculation programme (by the fundamental parameters method). The analysis was performed in the fluorine-uranium (F-U) range, and the contents of the elements thus determined were normalised to 100%.

The phase composition of the coal abrasives was studied using a Panalytical X’Pert PRO MPD X-ray diffractometer (PANalytical B.V., Almelo, The Netherlands). featuring a cobalt anode X-ray tube (λ_Kα_ = 0.179 nm) and a PIXcel 3D detector. The diffractograms were recorded in the Bragg–Brentano geometry with the angles ranging at 5–100° 2Theta with a step of 0.026° and a counting time of 80 s per step. Qualitative X-ray phase analysis was performed using the HighScore Plus (v. 3.0e) software and the PAN-ICSD dedicated database of inorganic crystal structures.

**Table 6:** Results of calculations of minimum oil film thickness h_min_ and relative oil film thickness λ for the roller–roller combination.

Parameters	Variant 0	Mean for Variant 1 ÷ 5
Minimum oil film thickness h_min_	0.212 μm	0.218 μm
Relative oil film thickness λ	0.445	0.456

**Table 7:** Results of calculations of Hertzian pressure and related parameters.

Hertzian pressure	420 N/mm^2^
Half pressure width b	0.074 mm
Maximum shear stress	126.3 N/mm^2^
Distance at maximum shear stress	0.058 mm

Spectroscopic analysis of the minerals tested was performed using an inVia Reflex Raman spectrometer from Renishaw featuring (Renishaw plc, Wotton-under-Edge, United Kingdom) a Leica Research Grade confocal microscope (Leica Microsystems GmbH, Wetzlar, Germany) capable of observing samples in reflected and transmitted light. Excitation was performed using a 50 mW ion-argon laser beam with a wavelength of λ = 514 nm. Sample measurements were recorded in a wide wave number range from 50 to 3200 cm^−1^.

The surface of the samples was observed using an Axia ChemiSEM (Thermo Fisher Scientific, Eindhoven, The Netherlands). scanning electron microscope (SEM) enabling secondary electron (SE) detection at an accelerating voltage of 20 kV and a magnification range of 60–1000×. The chemical composition of the test material in the micro-areas was subject to a qualitative analysis by energy dispersive X-ray spectroscopy (EDS) at an accelerating voltage of 20 kV. Prior to testing, the samples were coated with a thin film of gold to ensure electrical charge dissipation during the tests.

**Figure 6:**
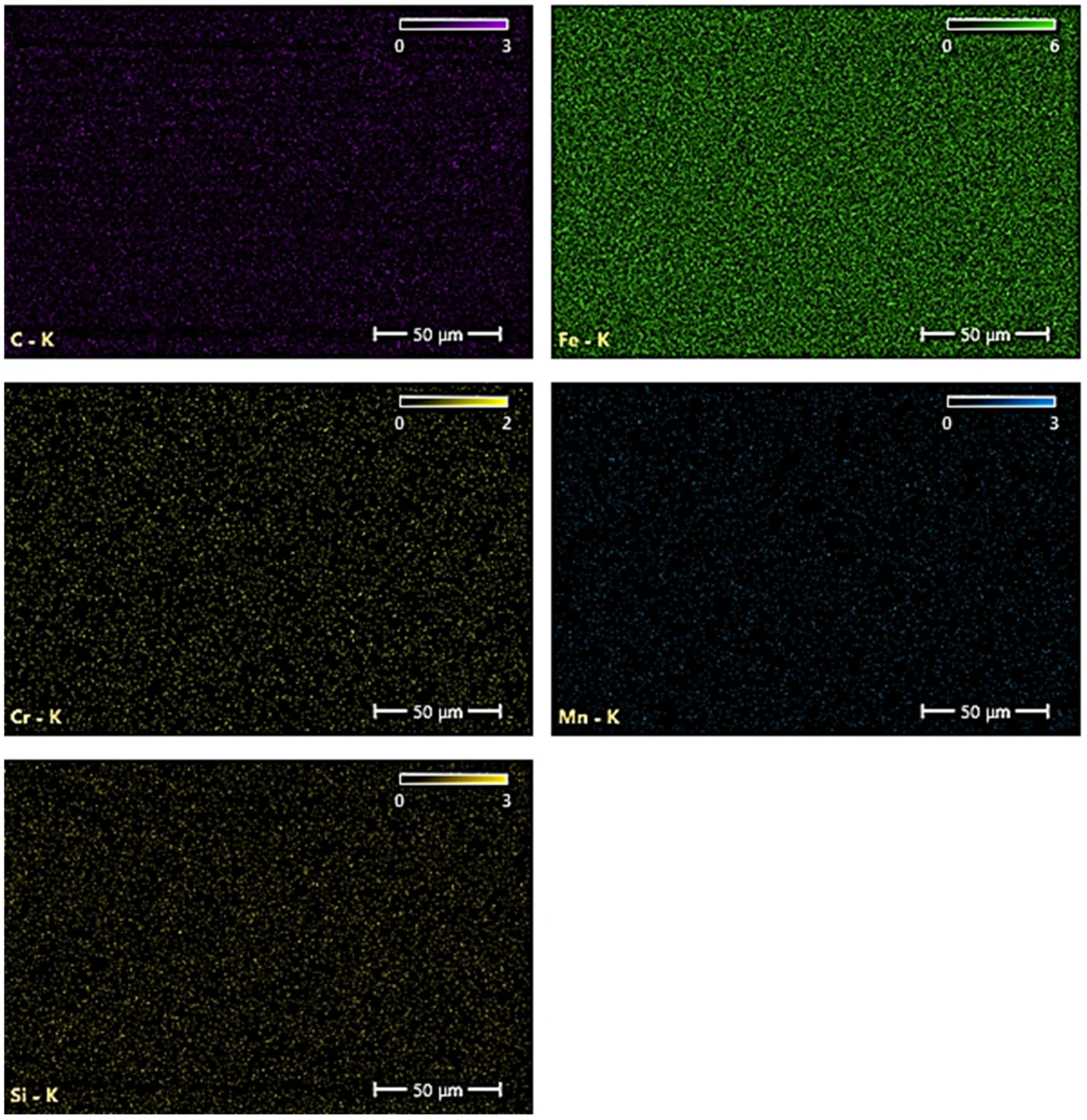
EDS elemental distribution maps of the sample surface before the test.

Figure 6 presents, for comparison purposes, the EDS maps of the elemental distribution on the surface of the samples before the test. The images show only a uniform distribution of the analysed elements, which is mainly related to their presence in the chemical composition of the 42CrMo-4 steel.

## 3. Results

### 3.1. Identification of the Abrasives Studied

In pursuit of the pre-defined objective of the study, namely identification of the quantitative and qualitative impact of hard coal and carbonaceous claystone-based oils on surface wear mechanisms, detailed identification of the mineral components of the mineral abrasives proved necessary.

Three coal-based abrasives (Variants 1–3) and two carbonaceous claystone-based abrasives (Variants 4–5) were used to test the wear of the lubricated friction nodes. Their chemical composition has been presented in Table 8. Based on the literature data [75], the hardness of hard coal was assumed to be approximately 572 MPa, while the hardness of coal shale was assumed to be about 690 MPa.

The coal abrasives subject to the tests were contaminated to varying degrees with mineral substance, the content of which was determined by the ash content in the coals, representing the residue left after combustion. The presence of the mineral substance in coal is attributable to the original plant-based material as well as the rock material penetrating the coal bed over the course of its formation (syngenetic components) or deposited at a later time (epigenetic components).

Generally, two types of the mineral substance can be distinguished:

– External substance, including minerals from the group of silicates, aluminosilicates, carbonates, sulphides, sulphates, and chlorides; this substance can be removed rather easily from coal by mechanical processing;
– Internal substance, which cannot be separated from the fuel by mechanical means; it consists of organometallic compounds, i.e., chemically bound metals with humic acids, waxes, and resins forming salts.

However, it should be noted that the ash obtained through the coal combustion differs in terms of quantity and quality from the mineral substance, subject to significant changes during the combustion, where one can observe phenomena such as decomposition of carbonates accompanied by CO_2_ release, loss of crystallisation water by silicates and aluminosilicates, etc. The ash content in coal varies from a few to even several dozen per cent, and such variation may be found between entire groups of deposits, between individual deposits, and even within deposits. The average ash content in coal is determined to range between 11 and 16 per cent. On account of the ash content, coal is broken down into low-ash (up to 20% ash) and high-ash (up to 40% ash) grades [76,77]. What has been found in the case of the abrasives studied is that Variant 1 represents low-ash coal, Variant 2—high-ash coal, while Variant 3, with the ash content exceeding 55%, may already represent a transitional form between coal and carbonaceous claystone. Due to the carbon content, Variant 1 should be classified as a bituminous coal of medium rank, whereas Variants 2 and 3 are considered low-rank coals (hard glossy lignites) [78]. The second group of samples is represented by carbonaceous claystones with an ash content exceeding 85%. One of the characteristics of coal-based claystones is that they contain carbonaceous substance in a dispersed form, referred to as pigment, which accounts for their distinctive black colour.

Having analysed further test results, one can conclude that the analysed coal abrasives (Variants 1–3) are characterised by a relatively low elemental C content, which typically ranges at 76–93% in hard coal [76,77]. For Variant 1, taking standard deviation into account, it can be assumed that the coal content falls within the standard, while for Variants 2 and 3, the coal content is 51.2% and 30.2%, respectively. This confirms the conclusion formulated above that Variant 3 is a transitional rock form between coal and carbonaceous claystone, which may also be a probable scenario for Variant 2, where the coal content barely exceeds 50%. These results correlate with the content of other components, where a significant increase in the SiO_2_ content can be observed in Variants 4 and 5 (50.06% and 57.42%, respectively), with a small fraction of silica in Variant 3 (31.16%). The results obtained for the content of Al_2_O_3_ and K_2_O are similar, indicating an increase in the fraction of argillaceous minerals in Variants 4 and 5.

**Figure 7:**
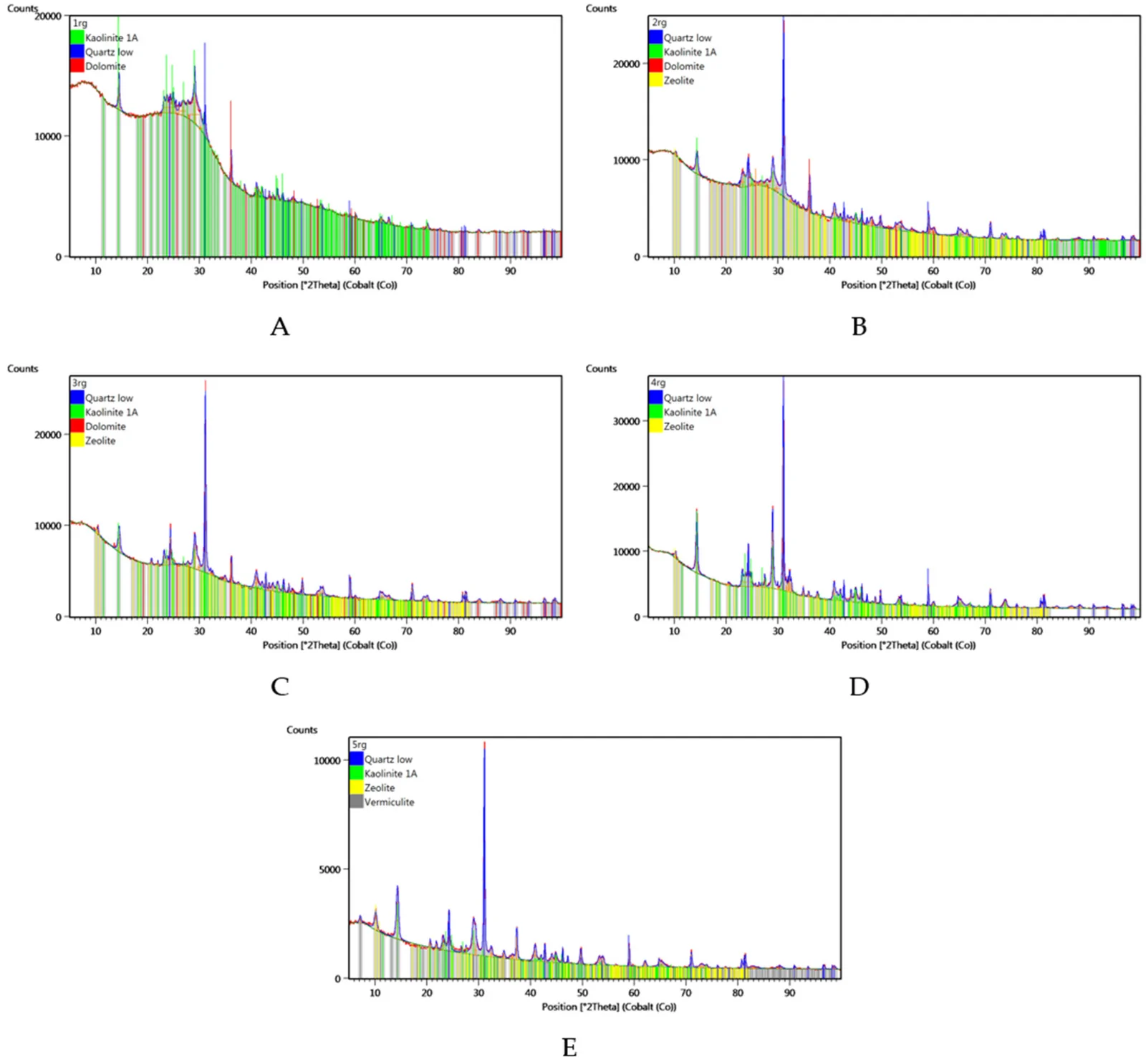
XRD test results for the abrasives studied: (**A**)—coal with ash content of λp = 9.24%; (**B**)—coal with ash content of λp = 29.15%; (**C**)—coal with ash content of λp = 55.28%; (**D**)—claystone with ash content of λp = 85.93%; (**E**)—claystone with ash content of λp = 89.78%.

In order to characterise the mineral components in a more detail manner, X-ray diffraction (XRD) tests were conducted (Figure 7). Based on the results thus obtained, the argillaceous minerals were found to be represented by kaolinite, while quartz and muscovite were also revealed to be present.

**Figure 8:**
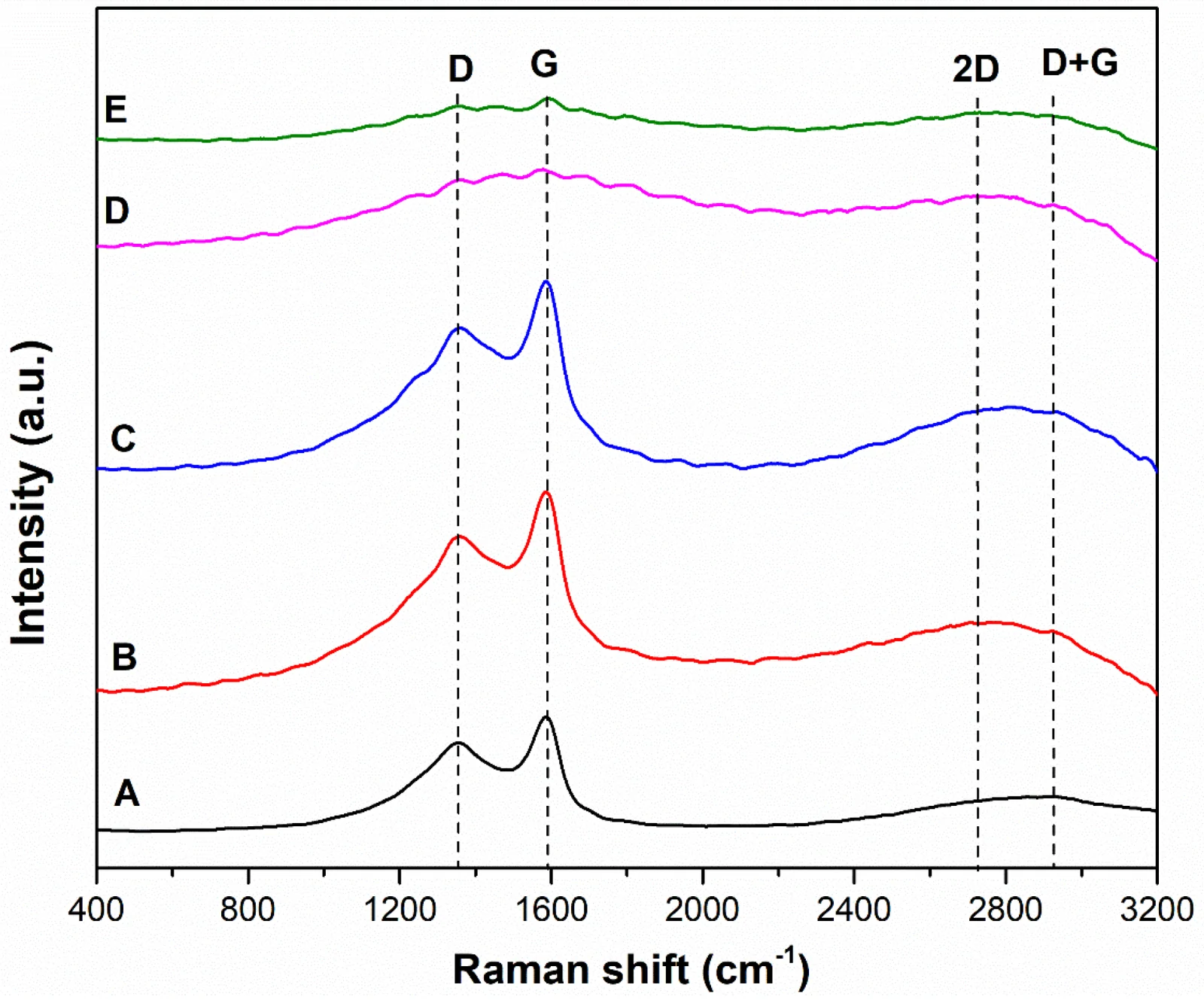
Raman spectra of the abrasives studied: (**A**)−coal with ash content of λp = 9.24%; (**B**)−coal with ash content of λp = 29.15%; (**C**)−coal with ash content of λp = 55.28%; (**D**)−claystone with ash content of λp = 85,93%; (**E**)−claystone with ash content of λp = 89,78%. D and G denote the characteristic disorder-induced (D) and graphitic (G) Raman bands of carbonaceous materials.

Additionally, the abrasives tested were structurally analysed using Raman spectroscopy. Figure 8 presents the Raman spectra for the individual types of abrasives studied. Four main modes (D, G, 2D, and D + G) can be distinguished in the spectra recorded, indicating the presence of amorphous carbon or highly disordered graphite in the structure of the materials tested. The vibrations of carbon skeletons (tensile vibrations in the C-C plane) characteristic of sp^2^ carbons correspond to the G mode, observed at ~1585 cm^−1^, while the occurrence of the D mode at ~1350 cm^−1^ indicates structural defects or disorder in the graphite lattice. The second-order D mode (2D) can be observed at ~2710 cm^−1^. Furthermore, the recorded D + G band (~2930 cm^−1^) implies a high degree of disorder in the carbon structure.

The Raman spectra analysis indicates that there is more carbonaceous material with a high degree of structural disorder (including graphite) in the first three samples, i.e., coal with an ash content of λ_P_ = 9.24% (material A in Figure 8), coal with an ash content of λ_P_ = 29.15% (material B in Figure 8), and coal with an ash content of λ_P_ = 55.28% (material C in Figure 8); they were compared with claystone with an ash content of λ_P_ = 85.93% (material D in Figure 8) and claystone with an ash content of λ_P_ = 89.78% (material E in Figure 8) [79,80].

Furthermore, in order to determine the degree of disorder in the carbon structure, the intensity ratio of the D band to the G band (I_D_/I_G_) was calculated for each of the investigated materials (Table 9). It can be observed that for materials A, B, and C, the I_D_/I_G_ ratio is similar and falls within the range of 0.75–0.78. In contrast, values of 0.90 and 0.85 were recorded for samples D and E, respectively. The pronounced increase in the I_D_/I_G_ ratio for the latter two materials (D and E) indicates a higher degree of structural disorder and a greater presence of structural defects compared to materials A, B, and C.

**Figure 9:**
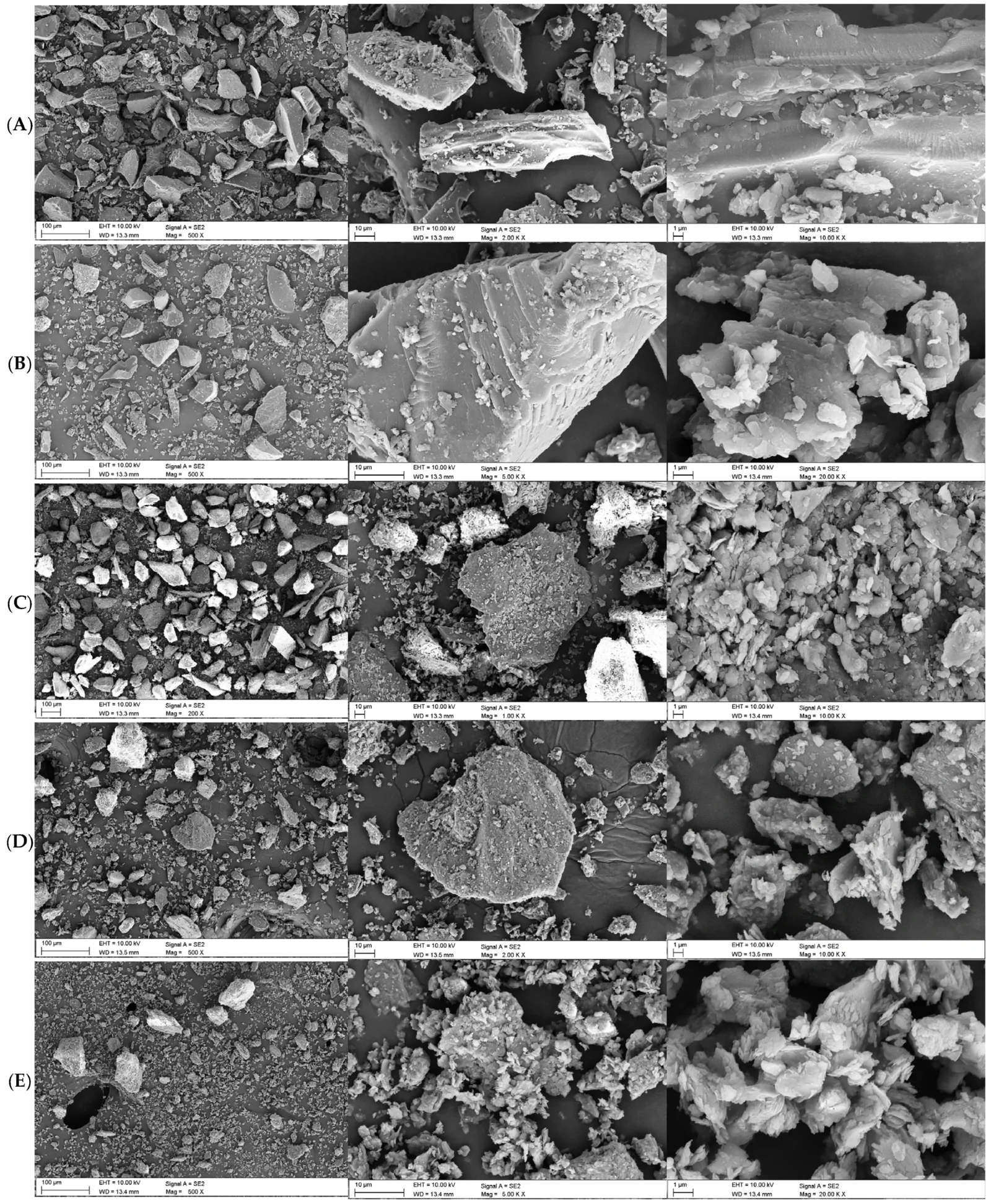
Grains of the abrasives studied (SEM image): (**A**)—coal with ash content of λp = 9.24%; (**B**)—coal with ash content of λp = 29.15%; (**C**)—coal with ash content of λp = 55.28%; (**D**)—claystone with ash content of λp = 85.93%; (**E**)—claystone with ash content of λp = 89.78% (For each abrasive, images are presented at three magnification levels).

Images of the grains of the abrasives tested, obtained by scanning electron microscopy (SEM), have been provided in Figure 9. Based on the aforementioned observations, it has been established that, regardless of the coal type analysed (Figure 6A–C), the grains presented are characterised by irregular shapes and varied sizes, falling within the pre-assumed grain size limits. However, grains larger than 25 µm predominate, accompanied by a significant fraction of finer particles. Larger grains are characterised by a heavily scratched surface, and one can also notice numerous grooves filled with finer material, which also accumulates on the surface of larger abrasive fragments. In the carbonaceous claystone-based abrasives, angular coal grains are generally visible alongside aggregates (clusters) of argillaceous minerals. The form in which they occur in this case is rather typical of these argillaceous minerals, which is related to the very small size of individual grains, usually having a flaky shape.

### 3.2. Wear Test Results

**Figure 10:**
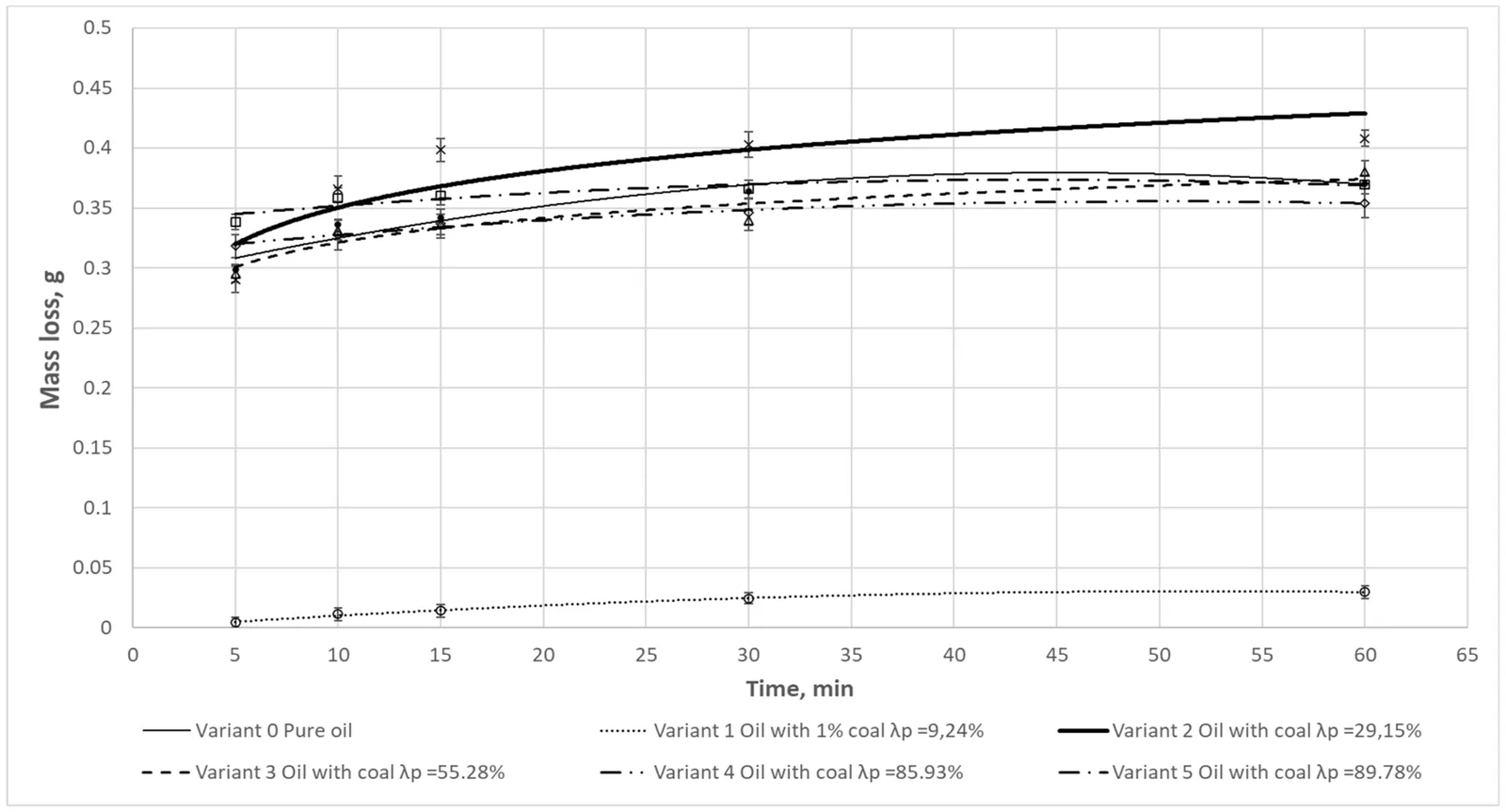
Mass loss determined for samples lubricated with oils containing carbonaceous contaminants in a function of time.

The results of the abrasive wear tests obtained for individual mixtures of oil with carbonaceous and argillaceous contaminants, in the form of mass loss curves in a function of time, have been provided in Figure 10. In the case of Variant 1, one can observe a slight effect of wearing-in on the mass loss value, while this effect is much more considerable in the other cases (Variants 0 and 2 ÷ 5). After the initial wear period (the first 5 min of the test), the surface of the samples tested under the conditions set for Variant 1 revealed shiny surface without any visible spalling under macroscopic magnification (approx. x30), and such a surface form remained throughout the test. In the case of the other variants, pits were clearly visible on the surface of the samples already after the first wear cycle, while the mass loss increments were relatively small.

**Figure 11:**
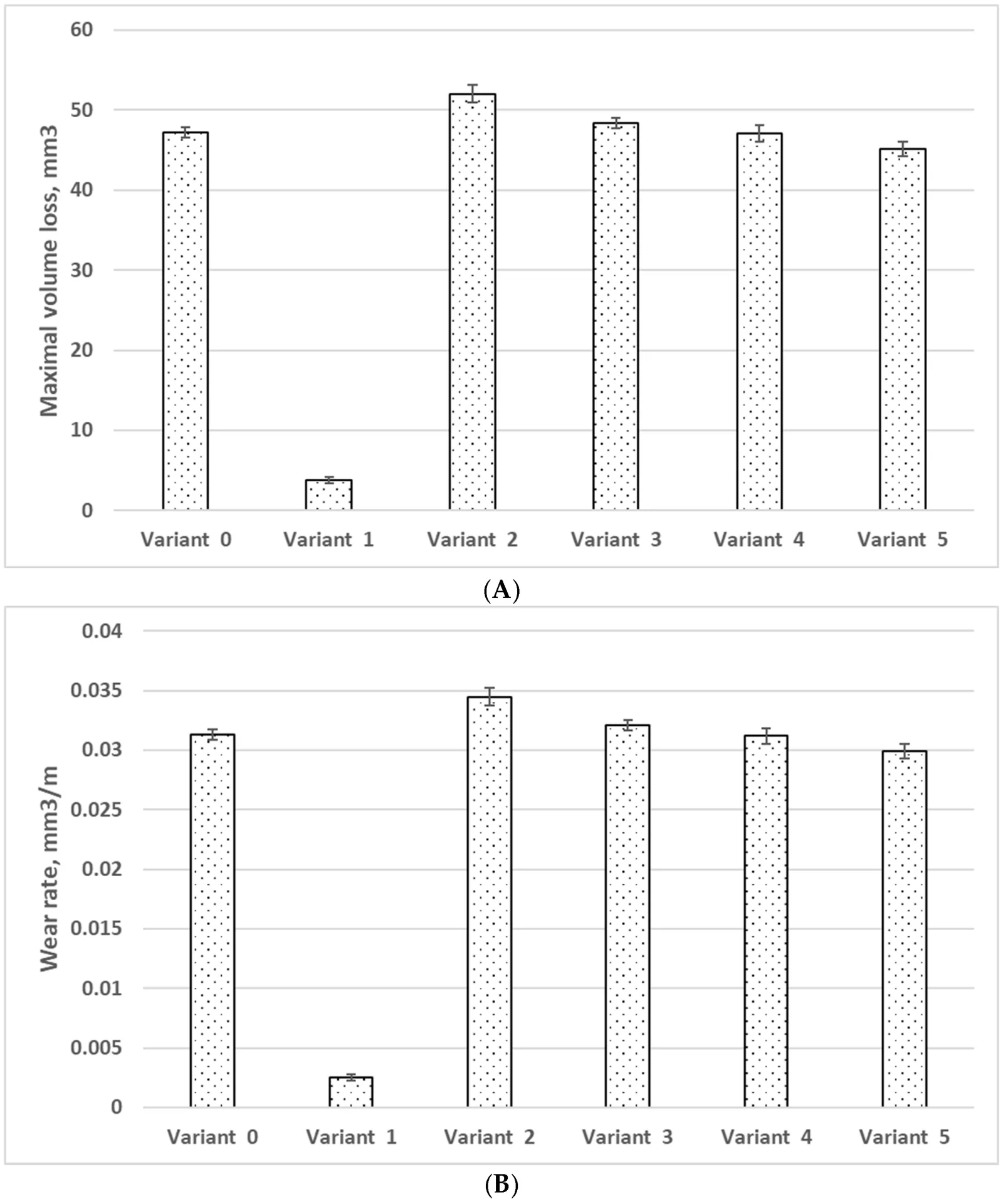
Summary of the maximum values of (**A**) volume loss and (**B**) wear rate for the tested variants.

Figure 11 shows a summary of the maximum values of volume loss and wear rate. The values obtained for Variant 0 and Variants 2–5 are similar to each other, similarly to the results presented in Figure 10. In contrast, the values determined for Variant 1 are on average approximately 12.5 times lower than those for the other variants, which clearly indicates its significantly improved anti-wear properties.

**Figure 12:**
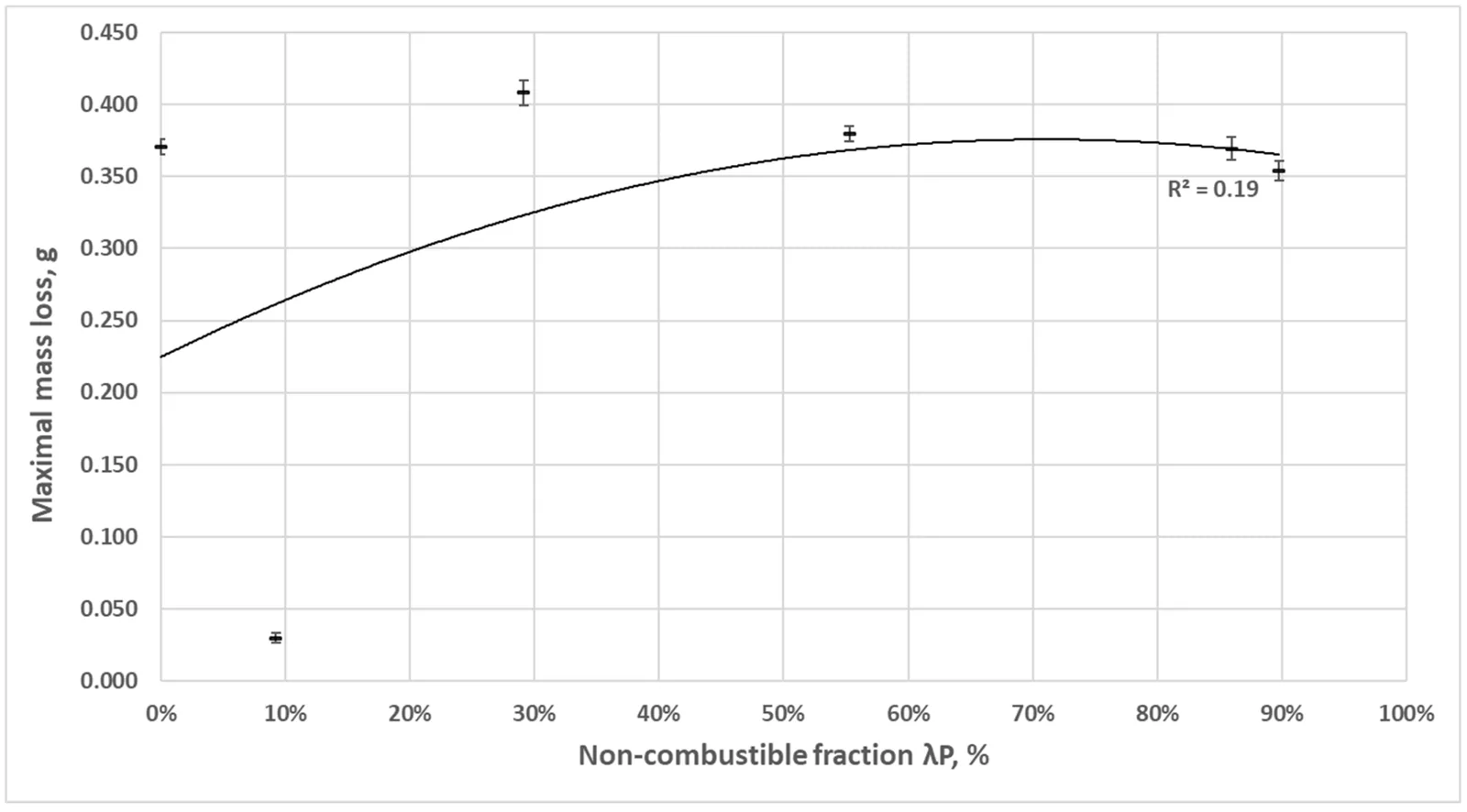
Maximum mass loss values determined for the variants studied and the corresponding percentage share of non-combustible fraction λ_P_ in carbonaceous contaminants.

Figure 12 shows the maximum mass loss values determined for the variants subject to tests in a function of the percentage share of non-combustible fractions λ_P_. Based on their values, the variants analysed can be ranked in the order of increasing wear: Variant 1 < Variant 5 < Variant 4 < Variant 0 < Variant 3 < Variant 2. What the figure also shows is that the wear determined for the case of lubrication with oil containing hard coal with the lowest ash content and the highest elemental carbon content (Variant 1) was characterised by the lowest wear, while the results obtained for the other variants, including the one with pure oil lubrication, were very similar (which is confirmed by the regression line determined for the results obtained for Variants 0 and 2 ÷ 5).

**Figure 13:**
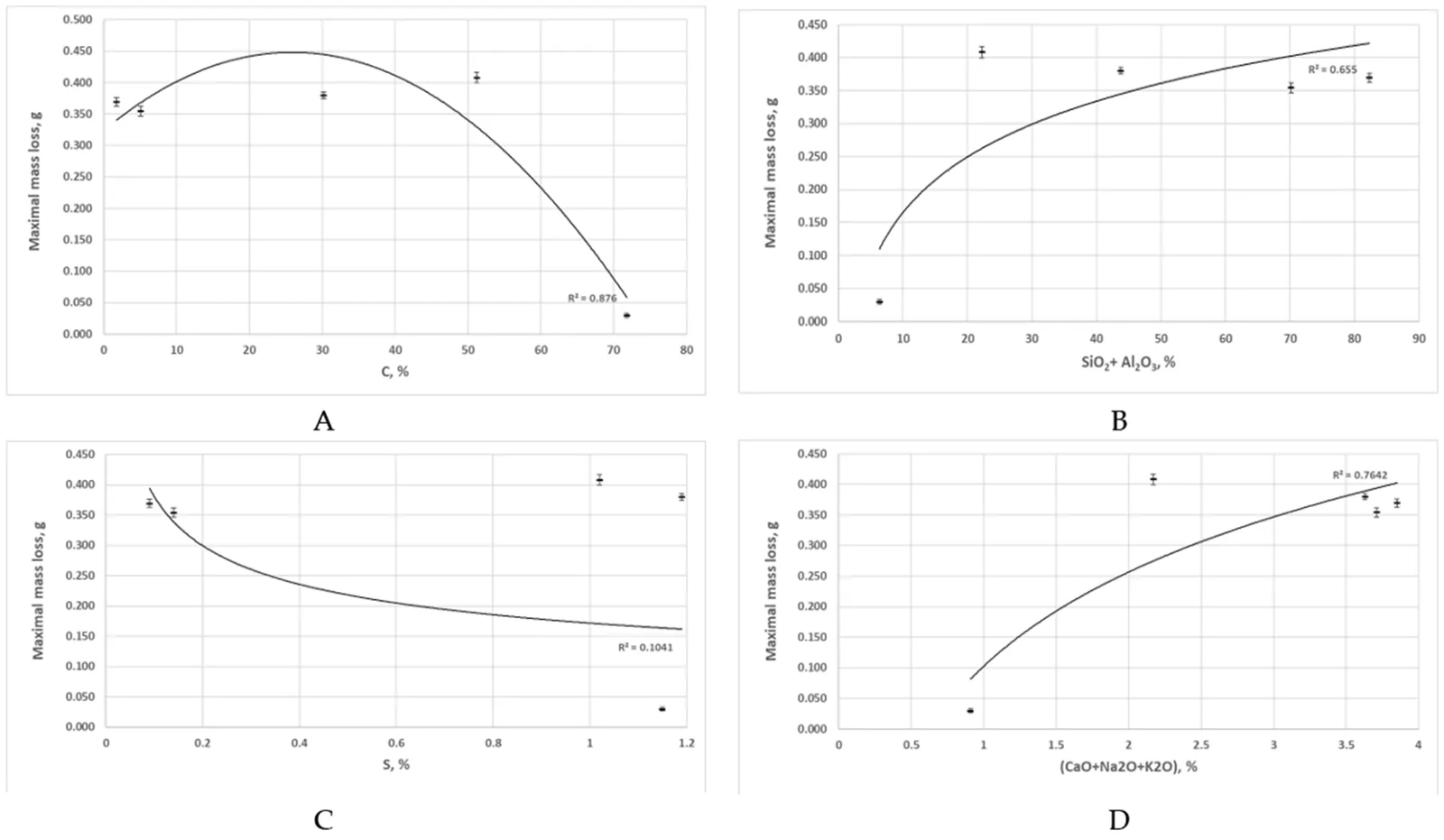
Effect of the share of individual mineral fractions in the abrasives based on hard coal and carbonaceous claystone on the mass loss in test samples: (**A**)—carbonaceous fraction, (**B**)—hard mineral fraction, (**C**)—sulphur, (**D**)—alkali metal oxide fraction.

Figure 13, on the other hand, provides graphs of the mass loss of the steel lubricated with oil contaminated with coal-mineral particles in a function of the mineral composition of the oil contaminants tested (cf. Table 7). The following fractions were analysed:

– Fraction of hard minerals (SiO_2_ + Al_2_O_3_), accountable for the abrasive properties of the materials examined,
– Carbonaceous fraction (C), determining the degree of carbonisation of abrasives,
– fraction of alkali metal oxides (CaO + Na_2_O + K_2_O), determining the capacity to form hydroxides with water,
– Sulphur determining the capacity to form superficial oxide layers.

What can be observed in the analysed range of the percentage share of non-combustible fractions λ_P_ = 9.24–89.78% in the carbonaceous contaminants of mineral oils is the mass loss declining as the degree of carbonisation of abrasives (Figure 13A) and sulphur content (Figure 13C) increase, as well as the mass loss increasing as the share of abrasive minerals, i.e., silica and aluminium oxide (Figure 13B), and the share of alkali metal oxide fractions (Figure 13D) increase.

### 3.3. Identification of Surface Damage Forms

Once the wear tests had been completed, the next stage in the research consisted in assessing the effects of the abrasive on the surface of the steel samples tested. To this end, microscopic observations of the surface of the samples were performed by scanning electron microscopy and light microscopy, and surface topography was measured using a digital confocal microscope.

**Figure 14:**
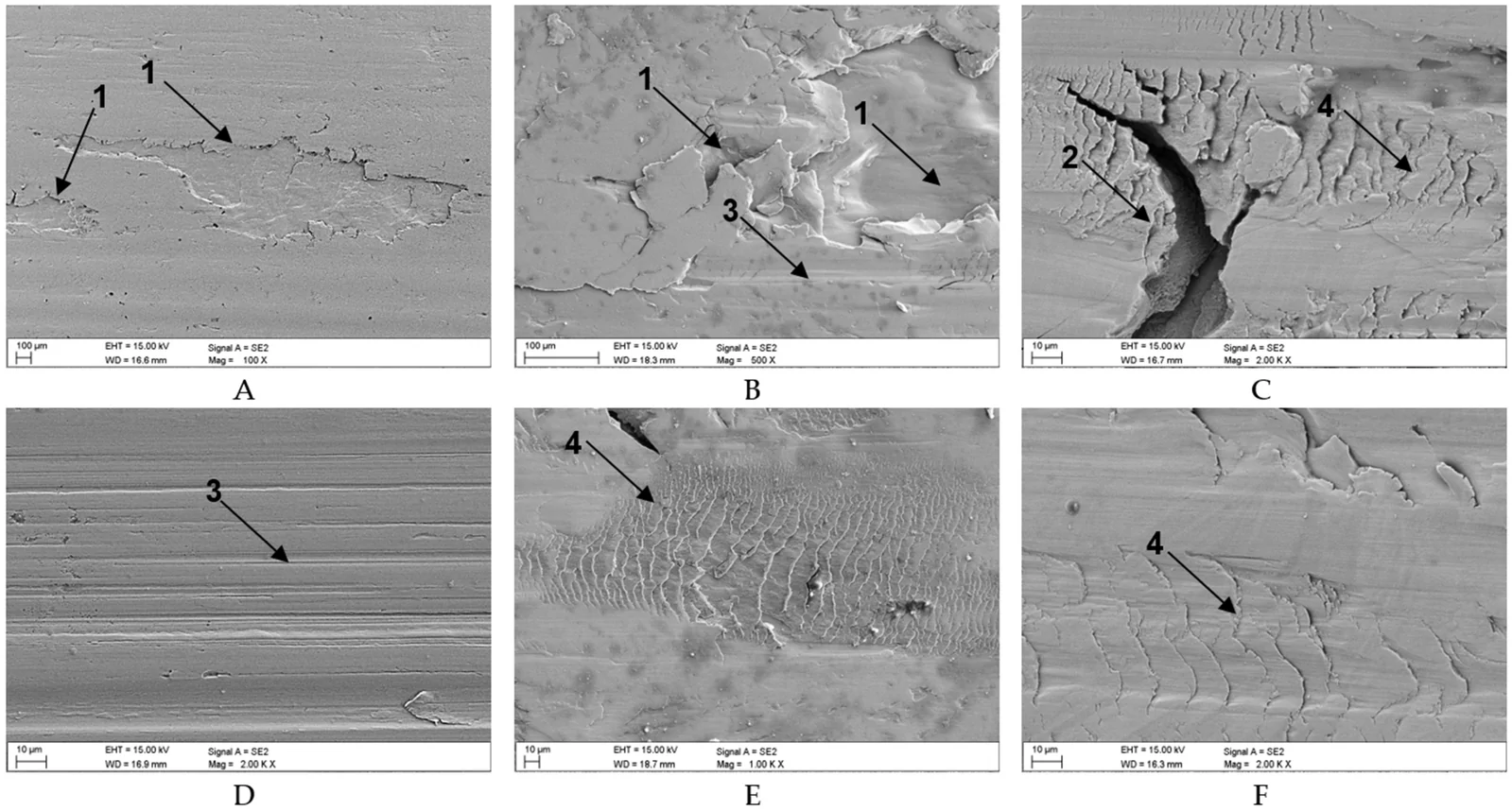
Images of the surface damage of the samples following wear tests in the presence of pure oil (Variant 0): (**A**,**B**)—surface spalling (delamination), (**C**)—surface cracking in the vicinity of pitting cracks, (**D**)—surface scratches, (**E**,**F**)—initial stages of pitting (SEM); Legend: 1—surface spalling, 2—surface cracking, 3—scratches, 4—initial forms of pitting.

Figure 14 provides images of the sample surface (lower and upper, respectively) lubricated with pure oil (Variant 0) following the wear test. It can be noticed that the dominant types of damage were scratches (Figure 14D) and flat surface spalling (Figure 14A,B). The presence of spalling indicates delamination under the impact of repeated load cycles caused by the movement of the abrasive material. The presence of scratches, on the other hand, implies the secondary impact of wear products on the surface of steel samples. What can also be observed in this case is surface texturing, suggesting the initial stages of pitting (Figure 14E,F), and surface cracking in the vicinity of parallel cracks (Figure 14C), which may herald delamination.

**Figure 15:**
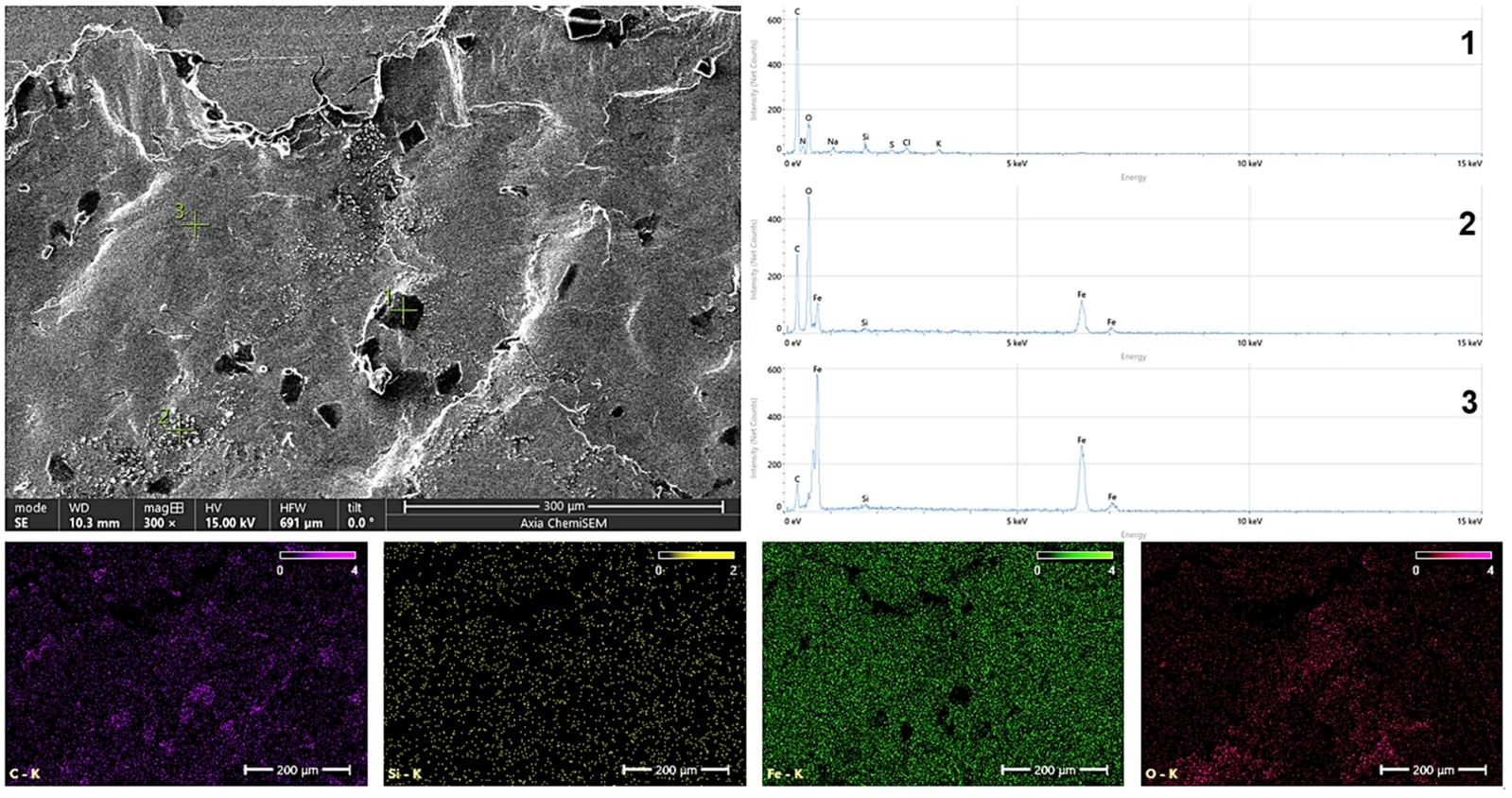
Surface view of the sample (SEM) obtained for Variant 0, EDS spectra for points 1, 2, and 3, and elemental distribution maps.

As shown in Figure 15, the SEM observations of Variant 0 were complemented by EDS spectra acquired at selected points and elemental distribution maps. The image shows the presence of carbon particles on the steel surface. In this variant, no additives or carbonaceous contaminants were introduced into the oil, indicating that the observed carbon was most likely formed as a result of thermal decomposition of the oil under conditions of friction and elevated contact temperature. This interpretation is further supported by the low silicon content revealed by the EDS analysis.

**Figure 16:**
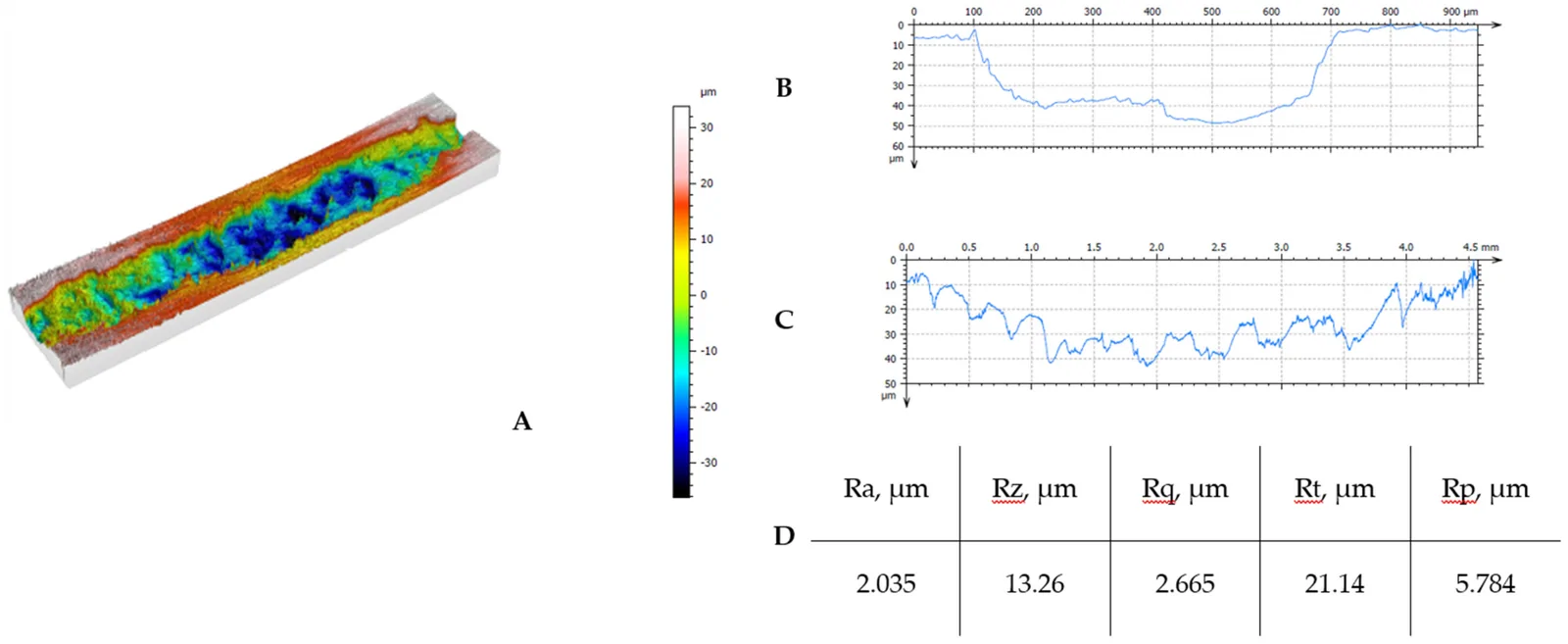
Results of profilometric tests of the surface of steel lubricated with pure oil (Variant 0) in the area of spalling: (**A**)−3D image of spalling, (**B**)−cross-section of spalling area, (**C**)−longitudinal section of spalling area, (**D**)−results of surface roughness measurements.

The steel surface profilogram provided in Figure 16 reveals extensive fatigue-induced delamination with a maximum depth of approx. 50 μm. The longitudinal section of the spalling area shows that the cracks spread at a slight angle into the surface layer, while the cross-section indicates that the spalling edges are almost vertical.

**Figure 17:**
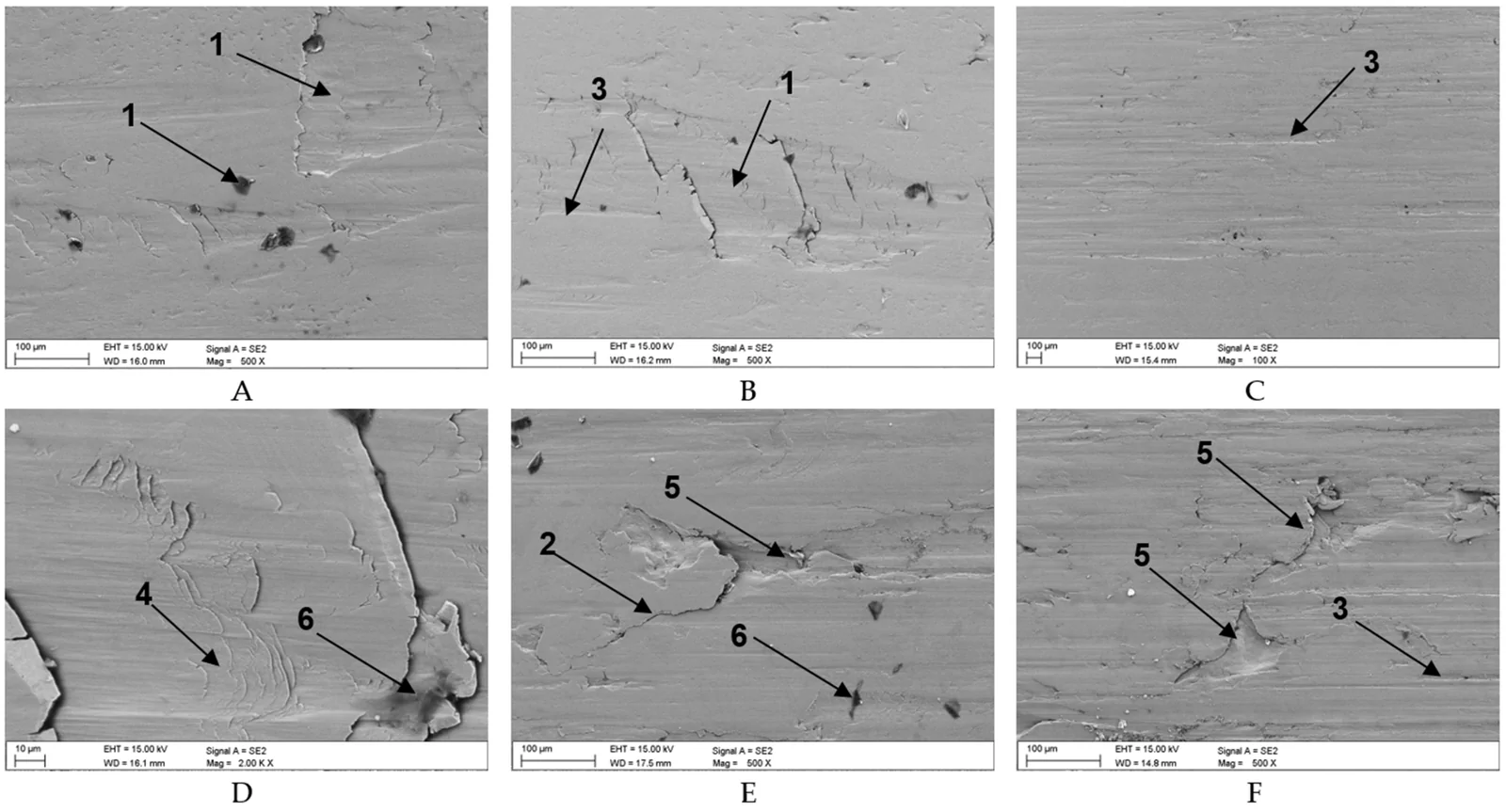
Sample surface defects following wear tests in the presence of a mixture of oil and coal with an ash content of λp = 9.24% (Variant 1): (**A**,**B**)—surface spalling (delamination) with visible fragments of flat coal grains, (**C**)—surface scratches with a small amount of coal grain, (**D**)—surface texturing typical of developing pits with a visible thin layer of coal, (**E**)—properly developed pitting phase with traces of coal particles, (**F**)—final pitting phase in the form of spalling (SEM); Legend: 1—surface spalling, 2—surface cracking, 3—scratches, 4—initial forms of pitting, 5—pitting, 6—coal.

Figure 17 provides images of the steel sample surface following the wear test in the presence of a mixture of oil and coal with an ash content of λp = 9.24% (Variant 1). The predominant types of damage identified for this variant were fatigue surface defects of flat spalling in the form of delamination platelets (Figure 17A,B) as well as the initial (Figure 17D,E) and final stages of pitting damage (Figure 17F). Shallow surface scratches with a few coal grains and pressed-in abrasive particles are also visible on the surface of the samples (Figure 17C).

**Figure 18:**
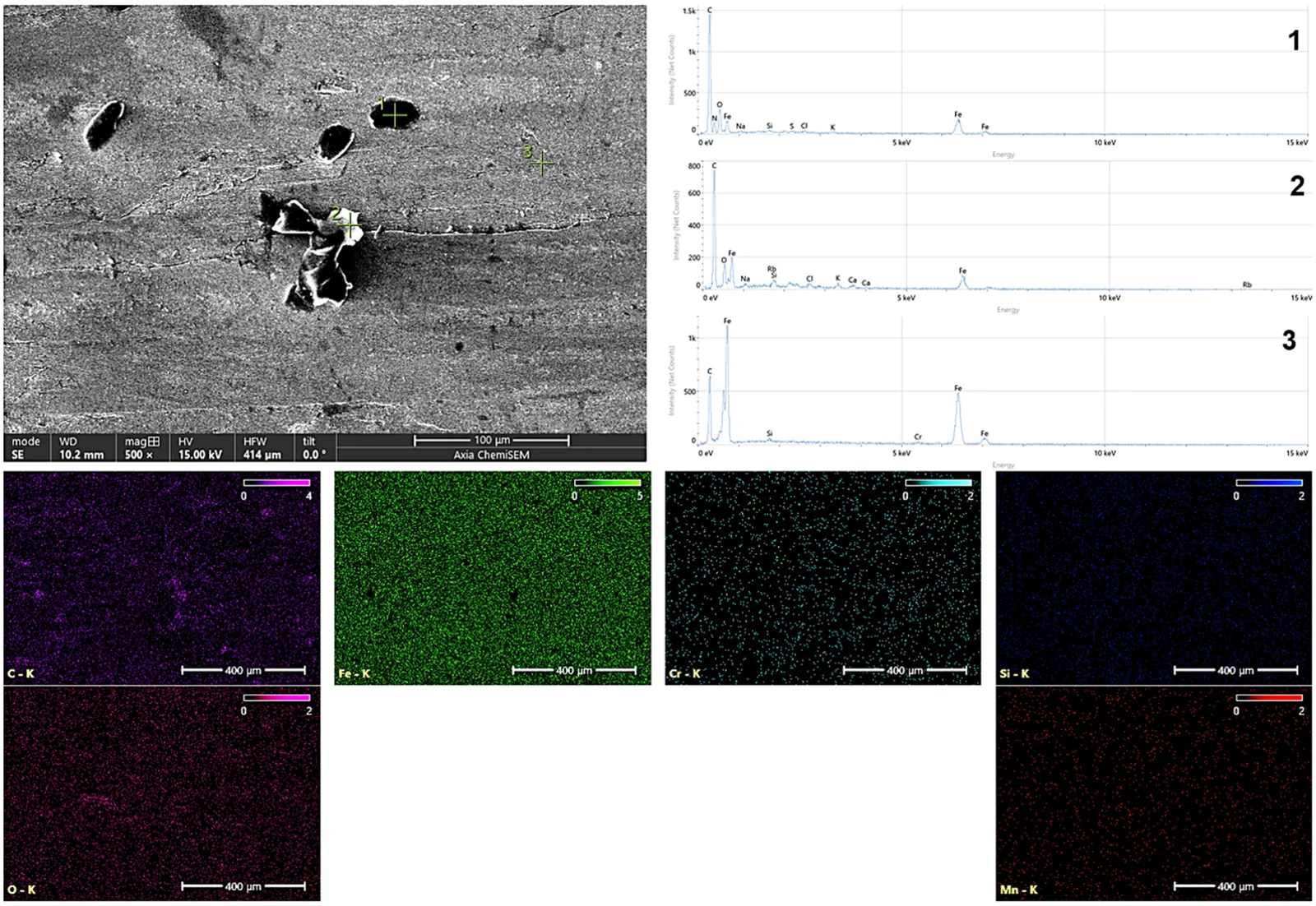
Surface view of the sample (SEM) obtained for Variant 1, EDS spectra for points 1, 2, and 3, and elemental distribution maps.

Figure 18 shows the results of energy-dispersive X-ray spectroscopy (EDS) analysis, which revealed two forms of carbon differing in chemical composition, indicating their distinct origins. In the first case (point 1), the carbon was formed as a result of the thermal decomposition of oil under conditions of friction and elevated contact temperature. In the second case (point 2), its presence resulted from the addition of carbon to the oil in the form of a contaminant. Furthermore, the carbon signal recorded at point 3, as well as its dispersion in the area outside the distinct carbon particles, indicates contact between the sample surface and carbon during tribological testing.

**Figure 19:**
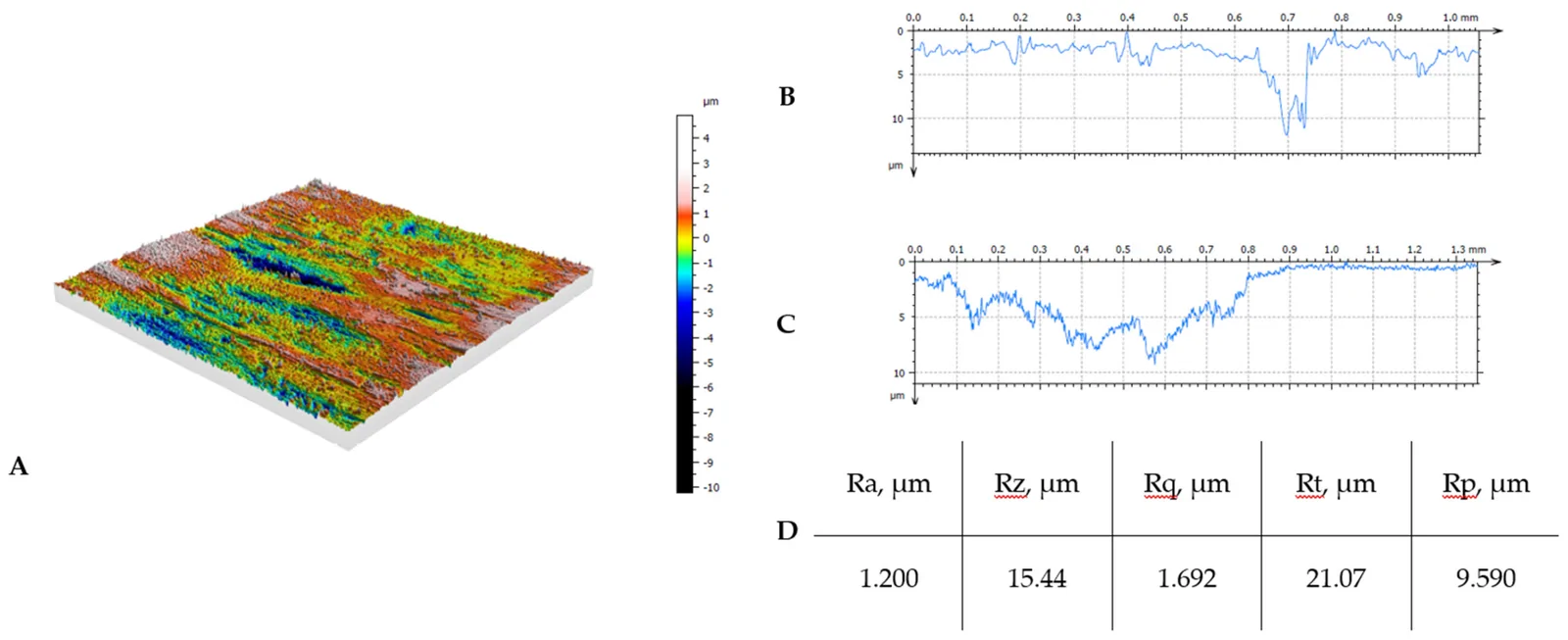
Results of profilometric tests of steel surface lubricated with oil contaminated with coal with an ash content of λp = 9.24% (Variant 1): (**A**)−3D image of spalling, (**B**)−cross-section of the spalling area, (**C**)−longitudinal section of the spalling area, (**D**)−results of surface roughness measurements.

The results of the profilometric tests conducted for Variant 1, as shown in Figure 19, illustrate a different form of spalling than that established for Variant 0, characterised by small shallow cracks with a maximum depth of approx. 12 μm.

**Figure 20:**
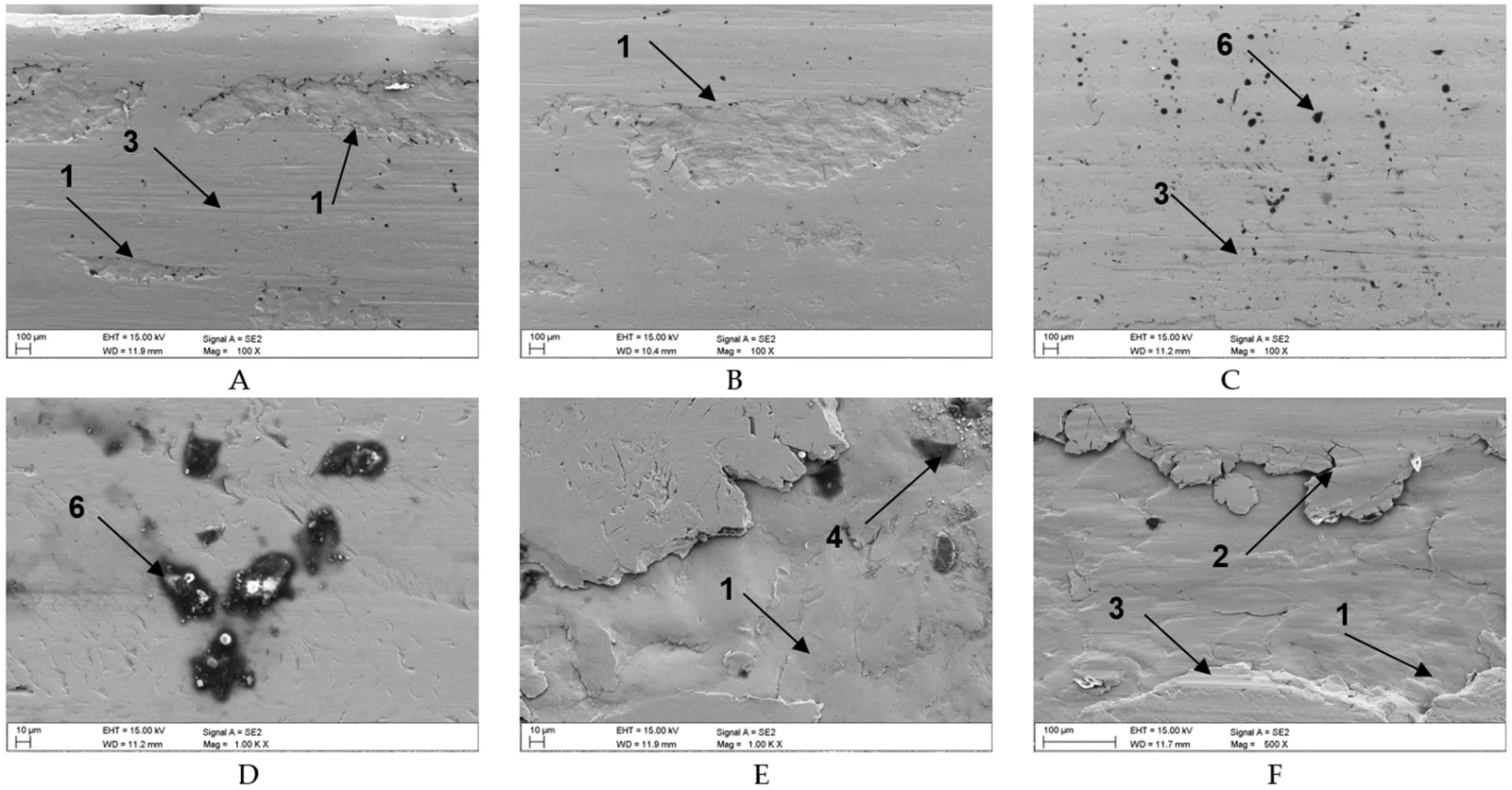
Sample surface defects following wear tests in the presence of a mixture of oil and coal with an ash content of λp = 29.15% (Variant 2): (**A**,**B**)—surface spalling (delamination) with visible fine coal grain fragments, (**C**)—surface scratches with numerous flat coal grains, (**D**)—fragments of superficial coal film, (**E**,**F**)—delamination edges with visible cracks and traces of coal particles (SEM); Legend: 1—surface spalling, 2—surface cracking, 3—scratches, 4—coal, 6—wear products.

**Figure 21:**
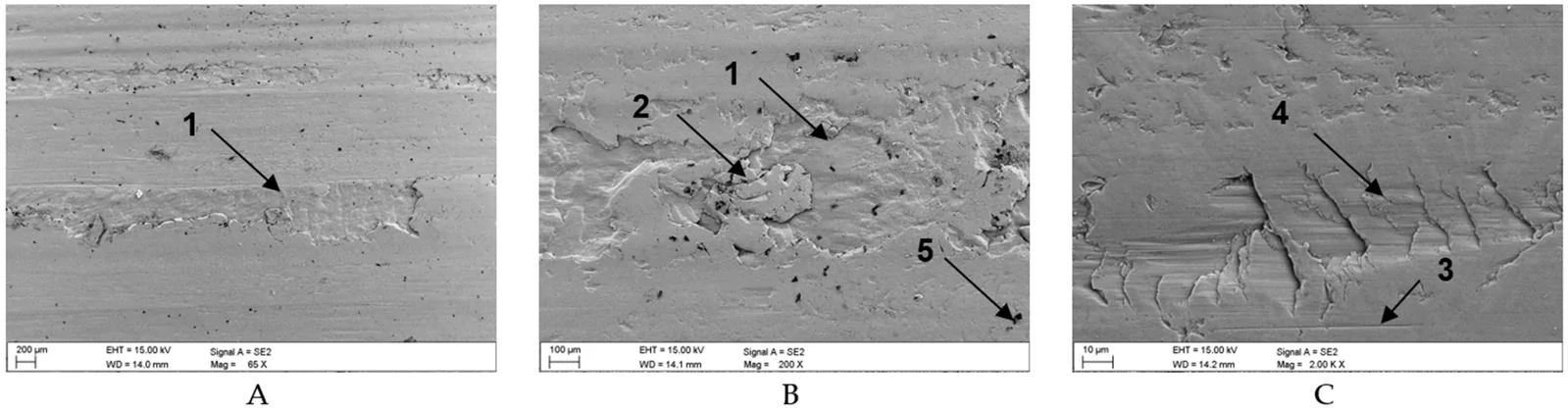
Sample surface defects following wear tests in the presence of a mixture of oil and coal with an ash content of λp = 55.28% (Variant 3): (**A**,**B**)—surface spalling (delamination) with visible small fragments of coal grains, (**C**)—initial and final phase of pitting with traces of surface scratches (SEM); Legend: 1—surface spalling, 2—surface cracking, 3—scratches, 4—initial forms of pitting, 5—coal.

**Figure 22:**
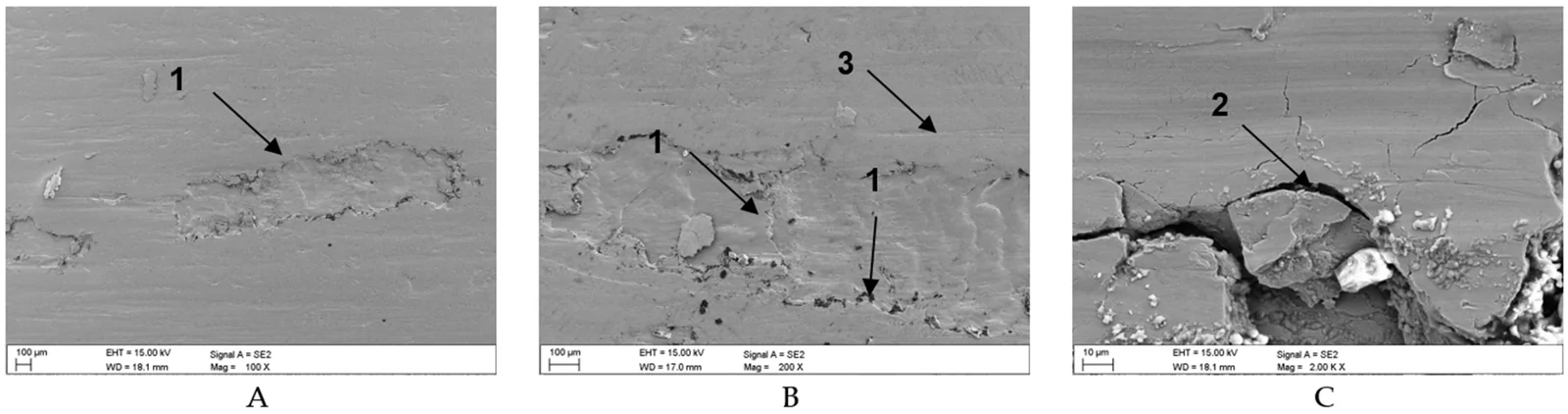
Sample surface defects following wear tests in the presence of a mixture of oil and coal with an ash content of λp = 89.78% (Variant 5): (**A**,**B**)—surface spalling (delamination) with visible small fragments of claystone arranged the edge, (**C**)—surface crack against the background of shallow surface scratches (SEM); Legend: 1—surface spalling, 2—surface cracking, 3—scratches.

Figure 20, Figure 21 and Figure 22 provide images of the surfaces of the samples lubricated with contaminated oil (Variants 2, 3 and 5, respectively) following the wear test (the surface view obtained for Variant 4 has not been provided due to its high similarity to that of Variant 5).

The predominant types of damage for all these variants were shallow scratches and flat surface spalls caused by surface delamination. Only for Variant 3 were there initial and final spalling marks typical of pitting. The forms of surface damage produced over the course of the wear tests under the conditions of lubrication with contaminated oil (Variants 2, 3, 4 and 5) were very similar to the defects identified when studying Variant 0. On the surfaces of these samples, embedded carbonaceous abrasive particles or products of thermal oil decomposition can also be observed, along with numerous artefacts exhibiting characteristics of corrosion products, mainly iron oxides.

**Figure 23:**
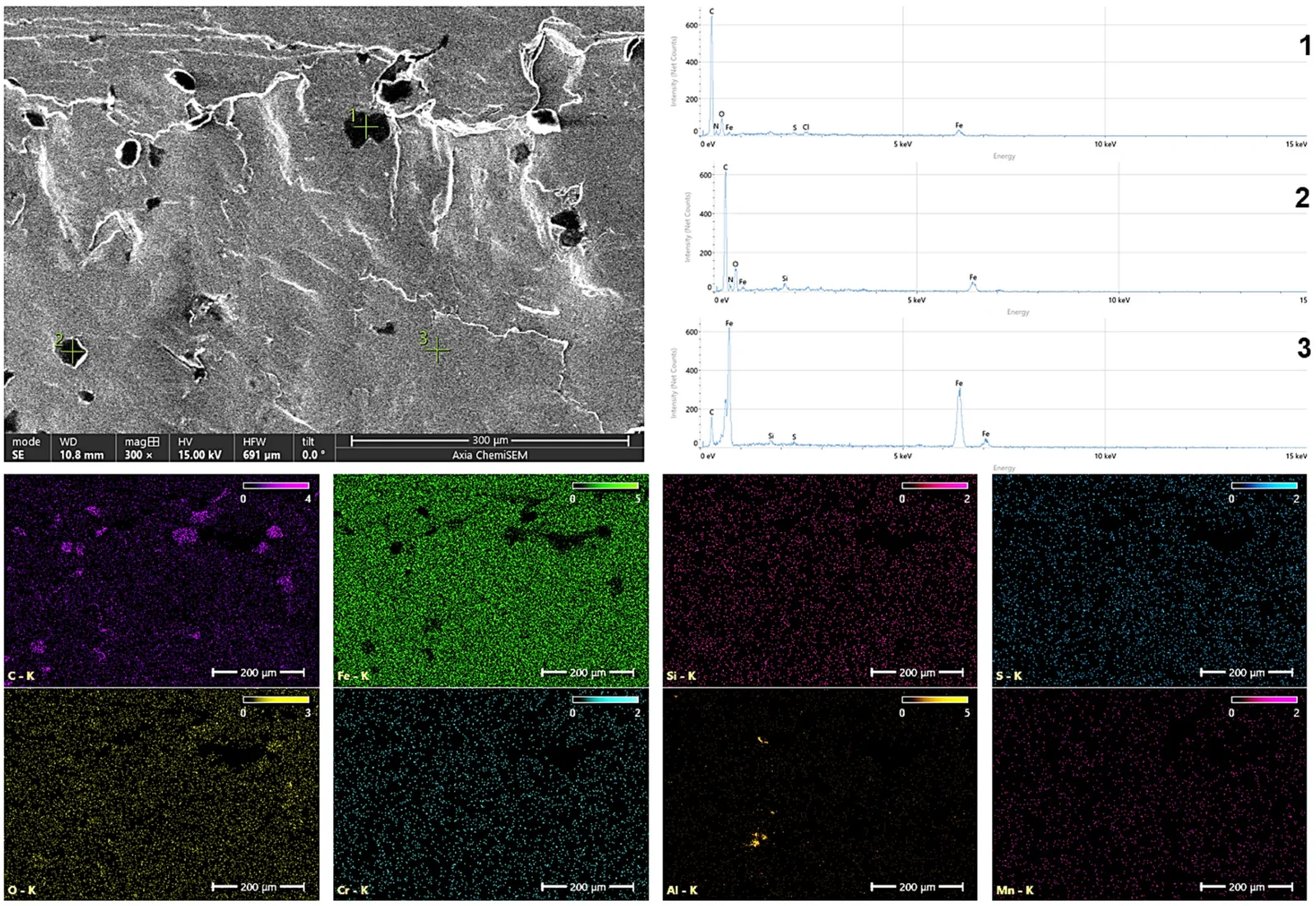
Surface view of the sample (SEM) obtained for Variant 5, EDS spectra for points 1, 2, and 3, and elemental distribution maps.

**Figure 24:**
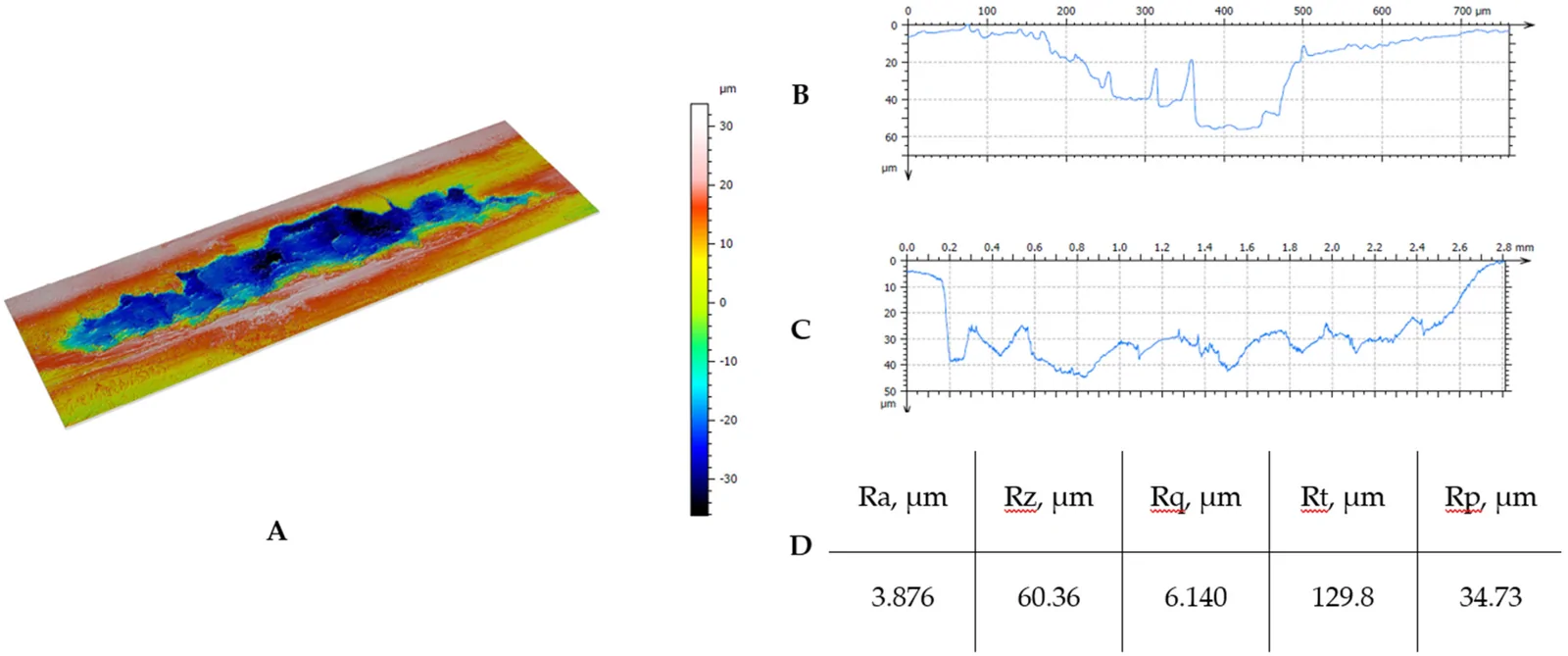
Results of profilometric tests of steel surface lubricated with oil contaminated with coal with an ash content of λp = 29.15% (Variant 2) in the spalling area: (**A**)−3D image of spalling, (**B**)−cross-section of the spalling area, (**C**)−longitudinal section of the spalling area, (**D**)−results of surface roughness measurements.

**Figure 25:**
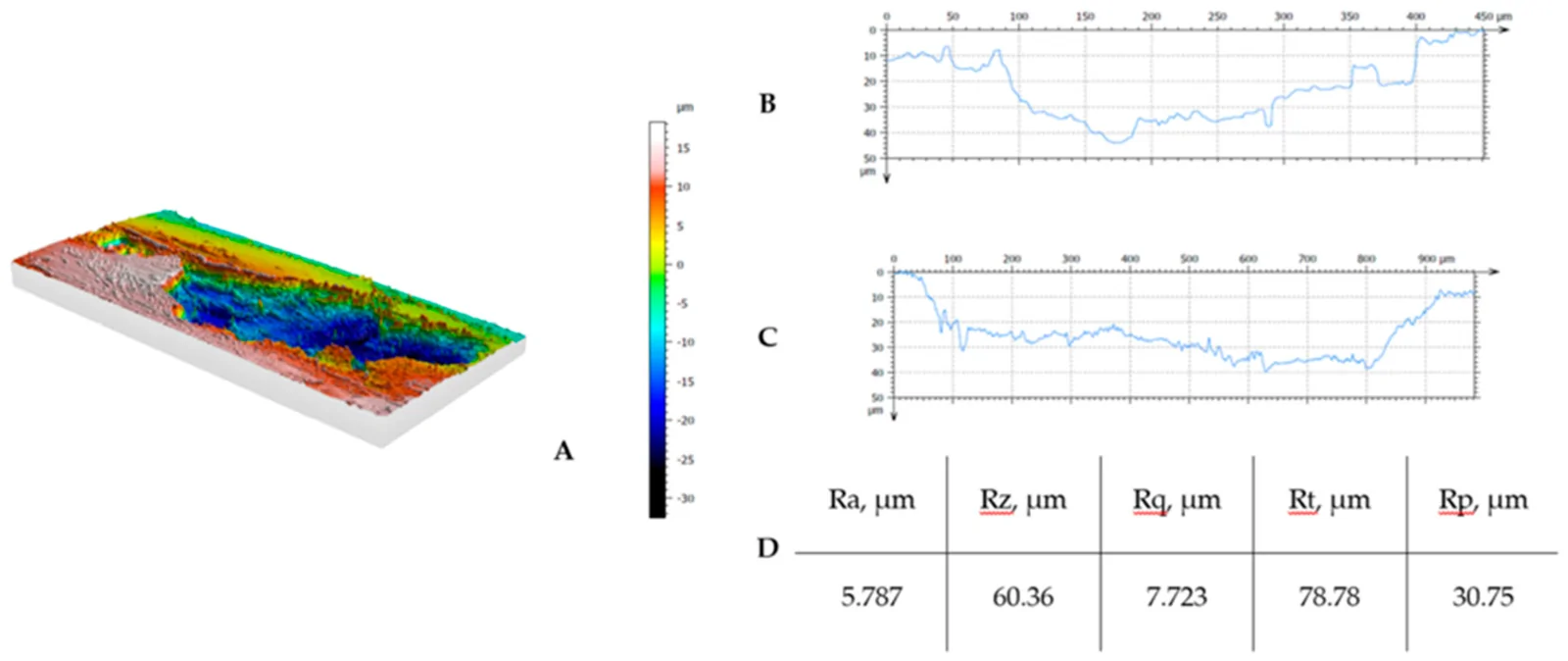
Results of profilometric tests of steel surface lubricated with oil contaminated with coal with an ash content of λp = 55.28% (Variant 3) in the spalling area: (**A**)−3D image of spalling, (**B**)−cross-section of the spalling area, (**C**)−longitudinal section of the spalling area, (**D**)−results of surface roughness measurements.

**Figure 26:**
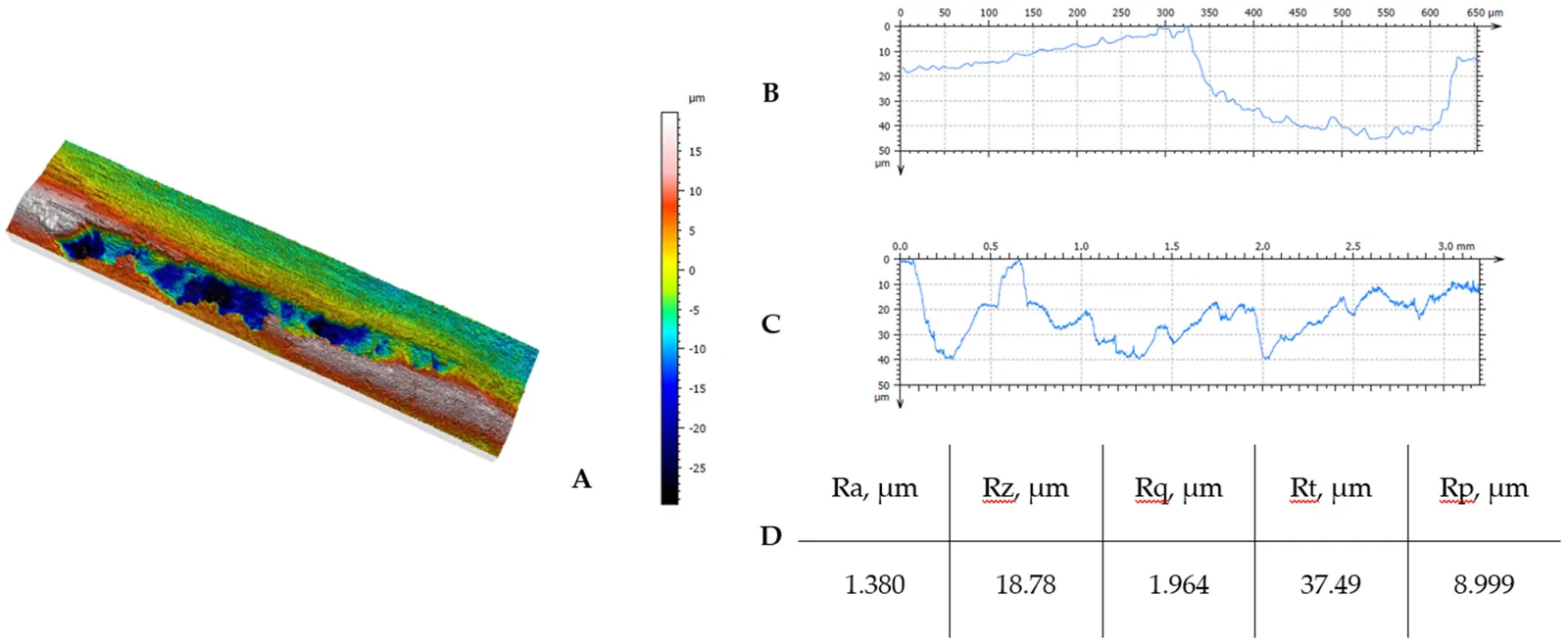
Results of profilometric tests of steel surface lubricated with oil contaminated with coal with an ash content of λp = 89.78% (Variant 5) in the spalling area: (**A**)−3D image of spalling, (**B**)−cross-section of the spalling area, (**C**)−longitudinal section of the spalling area, (**D**)−results of surface roughness measurements.

Figure 23, similar to the EDS analysis shown in Figure 19, reveals the presence of carbon particles of different origins, both resulting from the thermal decomposition of oil and from deliberately added carbon contaminants. A comparison of Figure 19 and Figure 23 indicates analogous mechanisms of surface modification and the formation of unstable carbon layers under the influence of friction and elevated contact temperature (see the following subsection). Similarly to the surface profilogram of the steel studied under Variant 0 (shown in Figure 16), the profilograms obtained for Variants 2, 3 and 5 (Figure 24, Figure 25 and Figure 26, respectively) reveal large fatigue-induced signs of delamination spalling which, however, differ slightly in terms of maximum depth: it is approx. 55 μm for Variant 2, while for Variants 3–5, it is approx. 45 μm.

The spalling depth values established for all the variants studied, save for Variant 1, are similar to those of the depth of maximum Hertzian stresses (cf. Table 6). The spalling forms identified for the variants employing the samples lubricated with coal-contaminated oil are also very similar to the damage found when studying Variant 0 (which also applies to Variant 4).

### 3.4. Calculation of the Flash Temperature in the Contact Area

EDS analyses indicate that, in variant 0, frictional interactions at elevated temperatures may have led to the formation of carbon, which can be associated with the thermal decomposition of the lubricant. To verify this observation, flash temperature calculations were performed using the Blok method [81]. The following relationship was used for the calculations:

(6) $$\Delta T_{flash}=\;\frac{1.11\cdot \mu \cdot W\cdot \left|U_{A}^{0.5}-U_{B}^{0.5}\right|}{{\left(2l\cdot 2b\right)}^{0.5\;}\cdot (K\cdot D\cdot c)}$$

where ΔT_flash_—the flash temperature in the contact zone (°C), μ—the coefficient of friction (-), W—the normal load acting on the contact (N), U_A,_ U_B_—the linear velocities of bodies A and B, (m·s^−1^), l—the half-length of the contact area (m), b—the half-width of the contact area (m), K—the thermal conductivity of the material (W·m^−1^·K^−1^), D—the material density (kg·m^−3^), c—the specific heat capacity of the material (J·kg^−1^·K^−1^).

The coefficient of friction (μ) was determined in the framework of study [47] using a pin-on-ring test rig for the same mixtures investigated in the present work. Table 10 presents the measured friction coefficients and the flash temperatures calculated from them using the Block method.

For the individual variants of the tested oil mixtures, the determined flash temperatures ranged from 449.7 to 518.4 °C (Table 10). High flash temperature values indicate the potential formation of thermal decomposition products due to friction arising from the combination of high contact pressure, significant sliding speed, and the limited thermal conductivity of the oil (K ≈ 0.13 W/(m·K)). A comparison of the calculated values with the flash temperatures reported by Nikas [65,66,67,68] showed good agreement, confirming the reliability of the applied calculation method.

In practice, the actual contact temperature may be slightly lower due to

– Heat conduction into the sample material,
– Convective heat transport by the oil,
– Heat dissipation resulting from dynamic oil replenishment.

## 4. Discussion

Having analysed Figure 12, which presents the maximum mass loss values as a function of the percentage share of non-combustible fractions λ_P_ in the carbonaceous contaminants, it can be observed that there is no clear functional relationship between wear and the ash content of the coal. However, a distinct local minimum in mass loss occurs at λ_P_ =9.24% corresponding to Variant 1. The differences in mass loss and wear rate among the remaining variants were considerably smaller.

Based on the characterisation of the investigated materials, including the data presented in Table 8, the material used in Variant 1 was found to have the highest degree of carbonisation among the materials analysed in this study. The degree of carbonisation affects the behaviour of carbonaceous material at elevated temperatures, including its susceptibility to plasticisation and the release of a tarry fraction at temperatures below 500 °C.

All solid fossil fuels, including hard coal, undergo thermal decomposition when heated under conditions of limited air access [82,83,84], leading to the release of liquid and gaseous products and the formation of a solid residue. The onset temperature of carbonaceous matter decomposition depends on the degree of carbonisation. This process may begin at approximately 170 °C for flame coals, 210 °C for gas coals, 300 °C for coking coals, and 320 °C for lean coals [85].

The intensive decomposition of the organic matter in coal occurs within the temperature range of 300–500 °C and begins at approximately 350 °C. Coals with an intermediate degree of carbonisation enter a plastic state within this temperature range. Liquid coal-derived products are then formed, including aromatic hydrocarbons with side chains, hydroaromatic hydrocarbons, higher phenols, and pyridine bases. This process is accompanied by the intensive release of tarry products. Approximately 70% of the tar is released at temperatures of up to approximately 400 °C, with the remainder being released at temperatures of up to approximately 500 °C. Above 500–550 °C, tar is no longer released [85].

According to one of the proposed mechanisms of coking coal carbonisation, depolymerisation reactions occur in the first stage, resulting in the formation of an unstable intermediate product known as metaplast, which causes the coal to become plasticised. Cracking reactions then occur, accompanied by the detachment of non-aromatic groups and the release of primary low-temperature carbonisation gas or primary tar. In the subsequent stage, condensation reactions of the metaplast units lead to the formation of a solid product, namely semi-coke [85].

The course of these transformations is influenced not only by the degree of carbonisation but also by the heating rate. During rapid pyrolysis, the yield of liquid coal-derived products is higher than during slow heating because the resulting liquid thermobitumens can be released before undergoing further decomposition. The release of primary tar may begin at a temperature as low as approximately 320 °C, with the onset temperature of this process depending on the degree of carbonisation [85].

Among the materials analysed in this study, only the material used in Variant 1 corresponded to the characteristics of coal susceptible to plasticisation within the temperature range occurring locally in the contact zone. The rapid heating of the particles, combined with their compression and shearing between counter-rotating surfaces, could therefore initiate transformations characteristic of the plasticisation of carbonaceous material, including the formation of metaplast and the release of a tarry fraction. These products could promote the compaction of particles and their adhesion to the steel surface, leading to the formation of a protective carbon-rich layer. This mechanism, rather than solely the carbon content or the similarity of its structure to graphite, may explain the different tribological behaviour of Variant 1.

For Variants 2–5, the oil mixtures contained carbon-mineral materials with an ash content of λ_P_ = 29.15–89.78%. As these particles passed through the contact zone, they could undergo compaction and thermal transformations. However, unlike Variant 1, no distinct protective effect that could be associated with the plasticisation of the carbonaceous material was observed. The resulting transformation products were probably brittle and may have occurred in the form of loose semi-coke grains and mineral particles involved in the surface wear process.

The presence of hard mineral fractions in the contaminants used in Variants 2–5 may have led to the formation of unstable layers of carbon-mineral material that did not effectively separate the interacting surface asperities under boundary friction conditions. Consequently, fragments of asperity peaks may have been detached, while the resulting particles contributed to surface scratching, as shown in Figure 20A, Figure 21A, and Figure 22B.

The analysis of the damage mechanisms showed that spalling of the surface layer was the dominant form of degradation of the 42CrMo4 steel surface. For Variant 1, the spalling depth was approximately 10 µm, whereas for the other variants it ranged from approximately 40 to 50 µm, corresponding to the location of the maximum contact stress zone (see Table 7). As shown in Figure 10, the greatest increase in mass loss, and thus the highest spalling intensity, occurred during the initial running-in phase of the samples. This stage was characterised by the most intensive thermal effects resulting from friction between surfaces with relatively high initial roughness. In the present study, wear tests were conducted on specimens with one specified initial surface roughness, which affected the lubrication conditions and the coefficient of friction. Based on the work of Polášek et al. [86], it should be stated that a change in surface roughness would most likely affect the wear intensity of 42CrMo4 steel.

The spalling observed after the wear tests for Variant 0 and Variants 2–5 was most likely caused by the combined action of high contact stresses, abrasive wear, and thermal stresses. This interpretation is consistent with both the spalling depth and the location of the maximum contact stress zone, as well as with the results of the flash-temperature analysis performed in Section 3.4 using Blok’s model.

It should be noted, however, that the friction coefficients used in the calculations were determined in previous studies using a pin-on-ring configuration, involving a rotating ring and a stationary block, and therefore under conditions that did not directly correspond to the counter-rotating motion of the two rings used in the present study. Nevertheless, the counter-rotating motion of the two specimens resulted in a considerably higher relative sliding velocity between the surface asperities, thereby promoting more intensive heat generation in the contact zone. It can therefore be assumed that using the friction coefficients determined in the pin-on-ring configuration did not result in an overestimation of the flash temperature, but rather provided a conservative estimate of the local thermal effects.

It should also be emphasised that the total contact surface temperature depends not only on the maximum flash-temperature rise but also on the bulk temperature of the materials before entering the contact, according to the following relationship:

(7) $$T_{c}=T_{b}+∆T_{flash}$$

where T_c_—maximum surface contact temperature, °C, T_b_—bulk temperature of the contacting solids before entering the contact, °C.

In the present study, the specimen temperature stabilised at approximately 60 °C. After accounting for this value, the maximum contact temperature T_c_, estimated using Equation (7), exceeded 500 °C.

Local temperatures exceeding 500 °C could have affected the properties of the surface layer of 42CrMo4 steel. However, due to the short duration of the flash temperatures, this effect cannot be unequivocally equated with the conventional high-temperature tempering process. Repeated heating and cooling cycles could instead generate local thermal stresses which, together with cyclic contact stresses, increased the susceptibility of the surface to crack initiation and propagation.

The compacted carbon-rich material could limit the direct interaction between asperities, thereby reducing the intensity of local heat generation and heat transfer to the steel substrate. This may explain why fully developed fatigue spalling in the form of pitting was not observed on the surfaces of the specimens tested under Variant 1 conditions, with only its initial forms being detected.

The decrease in wear intensity observed for Variants 2–5 with increasing coal ash content was most likely related to the tendency of clay mineral-rich particles to break down into finer grains (cf. Figure 6). According to the findings reported by Nikas [67,68], particle size, rather than particle hardness, has a decisive influence on flash temperature. Consequently, the susceptibility of clay particles to crushing could lead to a reduction in local temperature as they passed through the contact zone between the interacting surfaces.

For Variant 0, the effects of local surface heating were also observed, despite the absence of hard coal particles and mineral components in the lubricant. The spalling mechanisms and depths were similar to those observed for Variants 2–5. This indicates that the combined action of cyclic contact stresses and thermal stresses played an important role in the development of spalling, whereas the presence of solid contaminants modified the course of surface degradation.

EDS analysis revealed the presence of carbon-rich particles on the surfaces of the specimens tested under Variant 0 conditions. Since no hard coal or clay material was added in this variant, the presence of carbon can be associated with the thermal degradation of the lubricant in the contact zone. The calculated contact surface temperature exceeded the flash point of the tested oil, indicating the possibility of intensive local thermal transformations of the lubricant. A similar phenomenon was observed in [87], where carbon-rich deposits associated with the degradation of oil lubricating a 42CrMo4 steel ring gear were identified in a planetary gearbox operating under high mechanical and thermal loads. Moreover, the damage observed in an actual belt conveyor gearbox, including surface scratching, abrasive wear, local overheating, and tooth-surface spalling (Figure 1), indicates the practical susceptibility of 42CrMo4 steel gears to combined mechanical and thermal degradation under mining operating conditions. In contrast to the findings reported by Pang et al. [52], no characteristic molybdenum or phosphorus signals were detected that could indicate the presence of products derived from EP or AW additives. Only trace amounts of sulphur were detected in the EDS spectra.

## 5. Conclusions

Based on tests conducted on the wear of 42CrMo-4 steel under boundary friction conditions, lubricated with mineral oil containing carbon and clay impurities, the following conclusions were drawn:

1. The wear behaviour of 42CrMo4 steel lubricated with mineral oil contaminated by coal and claystone particles was governed by the combined influence of the degree of carbonisation, the chemical and mineralogical composition, and the resulting thermochemical behaviour of the contaminants under the conditions prevailing in the contact zone.
2. Variant 1, containing the material with highest carbon content and the lowest mineral fraction among the analysed carbonaceous materials, exhibited the lowest wear values of all tested variants. In particular, the maximum volume loss and wear rate were approximately 12.5 times lower than those observed for the other variants.
3. The favourable tribological performance of Variant 1 may be associated with the thermal and thermochemical properties of the carbonaceous material, resulting from its degree of carbonisation, particularly its susceptibility to plasticisation and the formation of liquid coal-derived products during rapid heating. These products could promote particle compaction and adhesion to the steel surface, leading to the formation of a protective carbon-rich layer that limited direct interaction between surface asperities.
4. The depth of surface observed spalling for Variant 1 was approximately 10 µm, which corresponds to roughly 25% of the spalling depth observed for the other variants (Variants 0 and 2–5).
5. Calculations using Blok’s model indicated that the flash-temperature rise, $\Delta T_{flash}$, ranged from 449 to 518 °C. After accounting for the bulk specimen temperature of approximately 60 °C, the estimated maximum contact surface temperature exceeded 500 °C. These results support the possibility of thermal degradation of the lubricant and thermochemical transformations of the carbonaceous material in the contact zone, but do not constitute direct evidence of these processes.
6. In Variants 2–5, the carbonaceous and clay-rich materials did not exhibit a protective effect comparable to that observed for Variant 1. Their lower degree of carbonisation and higher mineral content may have limited their susceptibility to plasticisation, while their thermal transformation could have resulted in the formation of brittle solid products that contributed to surface wear.
7. EDS analysis revealed carbon-rich particles on the surfaces tested under Variant 0 conditions, indirectly indicating thermal degradation of the mineral oil and high local contact temperatures.
8. Lubrication with pure oil did not fully prevent direct interaction between surface asperities. The extensive surface spalling observed for Variant 0 may have resulted from the combined action of cyclic contact stresses and local thermal stresses.
9. The results indicate that the wear behaviour of the steel surfaces was affected by the combined influence of the degree of carbonisation, the chemical and mineralogical composition of the contaminants, and the resulting thermochemical behaviour of the carbonaceous material under the conditions prevailing in the contact zone.

## Figures and Tables

**Table 1:** Concentration, type, and size of particles in lubricating oil, retrieved from different operating environments; based on [35].

Oil Sampling Location	Concentration, g/dm^3^	Grain Size Range, 10^−6^ m	Contamination Type	Source
Paper mill	0.4	0 ÷ 150	Cu, Si, Silicates	[36]
Motor vehicle sump	1 ÷ 2	0 ÷ 250	C, Fe	[36]
Various bearing lubricants	0.3 ÷ 1.5	0 ÷ 250	Fe, Al, Cu, Sn, SiC, sand	[37]
Various		0 ÷ 100	Al, Ag, Cr, Zn, Fe	[38]
Motor vehicles	0.3	0 ÷ 120	Al, Cu, Fe, Pb	[38]
Aircraft gas turbines	1 ÷ 2	0 ÷ 200	Fe, C, silicates	[39]

**Table 2:** Theoretical maximum flash temperatures during ductile debris extrusion in a typical line, rolling-sliding, elastohydrodynamic contact. Counterface hardness is 800 HV. (Adapted from the work of Nikas [65,66]).

**Particle Diameter (µm)**	**Particle Hardness (HV)**	**Maximum Flash Temperature (°C)**	
		**Surface 1**	**Surface 2**
5	100	93	17
5	200	185	34
5	400	350	68
10	100	211	103
10	200	415	182
10	400	760	318
20	100	1350	846
20	200	1560	962
20	400	1830	1120

**Table 3:** Chemical composition of the 42CrMo-4 steel [mass%].

**C**	**Si**	**Mn**	**S**	**P**
0.343 ± 0.012	0.54 ± 0.02	1.07 ± 0.03	0.014 ± 0.002	0.007 ± 0.001
**Cr**	**Cu**	**Ni**	**Mo**	**Al**
0.433 ± 0.01	0.0729 ± 0.004	0.418 ± 0.02	0.235 ± 0.01	0.0639 ± 0.004

**Table 4:** List of lubricating oil mixtures as well as coal and coal-mineral contaminants examined.

Variant No.	Oil Tested	Mass Fraction of Abrasive, %	Grain Size Range
0	Class VG 220 mineral oil	0	
1		1	0−100
2			
3			
4			
5			

**Table 5:** List of parameters characterising clean and contaminated lubricating oil.

Parameters	Variant 0	Mean for Variant 1 ÷ 5
Kinematic viscosity at 40 °C	189 mm^2^/s	195.4 mm^2^/s
Kinematic viscosity at 100 °C	16.42 mm^2^/s	16.6 mm^2^/s
Density at 15 °C	880 kg/m^3^	890 kg/m^3^
Flash point	260 °C	-

**Table 8:** Chemical analysis of test samples.

Component, wt.%	Variant No.				
	1	2	3	4	5
C, %	71.80 ± 2.90	51.20 ± 2.10	30.20 ± 1.20	5.08 ± 0.51	1.75 ± 0.18
S, %	1.15 ± 0.17	1.02 ± 0.15	1.19 ± 0.18	0.14 ± 0.05	0.09 ± 0.04
SiO_2_, %	3.72 ± 0.19	15.75 ± 0.79	31.16 ± 0.78	50.06 ± 1.25	57.42 ± 1.44
Al_2_O_3_, %	2.65 ± 0.13	6.50 ± 0.32	12.62 ± 0.63	20.13 ± 1.01	24.90 ± 1.24
Fe_2_O_3_, %	1.10 ± 0.11	1.90 ± 0.19	3.99 ± 0.20	8.87 ± 0.44	2.29 ± 0.11
TiO_2_, %	0.10 ± 0.05	0.30 ± 0.15	0.55 ± 0.27	0.83 ± 0.42	0.58 ± 0.29
MnO, %	0.02 ± 0.01	0.03 ± 0.02	0.10 ± 0.05	0.22 ± 0.11	0.03 ± 0.01
CaO, %	0.57 ± 0.28	1.36 ± 0.14	1.69 ± 0.17	0.66 ± 0.33	0.13 ± 0.06
MgO, %	0.31 ± 0.15	0.94 ± 0.47	1.57 ± 0.16	1.94 ± 0.19	0.85 ± 0.42
Na_2_O, %	0.20 ± 0.10	0.08 ± 0.04	0.23 ± 0.11	0.26 ± 0.13	0.94 ± 0.47
K_2_O, %	0.14 ± 0.07	0.73 ± 0.36	1.71 ± 0.17	2.79 ± 0.14	2.78 ± 0.14
P_2_O_5_, %	0.10 ± 0.05	0.03 ± 0.02	0.13 ± 0.06	0.22 ± 0.11	0.07 ± 0.04
Ash, %	9.24% ± 0.51	29.15% ± 0.51	55.28% ± 0.51	85.93% ± 0.51	89.78% ± 0.51

**Table 9:** Values of the D and G band parameters and the corresponding I_D_/I_G_ ratios determined from the recorded Raman spectra.

Sample	ω_D,_ cm^−1^	ω_G,_ cm^−1^	I_D_/I_G_
coal with ash content of λp = 9.24% (A)	1363	1587	0.76
coal with ash content of λp = 29.15% (B)	1356	1587	0.78
coal with ash content of λp = 55.28% (C)	1360	1582	0.75
claystone with ash content of λp = 85.93% (D)	1351	1579	0.90
claystone with ash content of λp = 89.78% (E)	1344	1591	0.85

**Table 10:** Friction coefficients and flash temperatures determined using the Block method for the tested mixtures.

	Variant 0	Variant 1	Variant 2	Variant 3	Variant 4	Variant 5
μ	0.094 ± 0.002	0.093 ± 0.001	0.095 ± 0.001	0.097 ± 0.001	0.085 ± 0.002	0.098 ± 0.001
ΔT_flash_	497.3 ± 10.6	488.8 ± 5.3	502.6 ± 5.3	513.2 ± 5.3	449.7 ± 10.6	518.4 ± 5.3

## Data Availability

The original contributions presented in this study are included in the article. Further inquiries can be directed to the corresponding author.
